# Nanotherapeutic Formulations for the Delivery of Cancer Antiangiogenics

**DOI:** 10.1021/acs.molpharmaceut.4c00822

**Published:** 2025-04-04

**Authors:** Amelia Ultimo, Ayushi Jain, Elisabet Gomez-Gonzalez, Thomson Santosh Alex, Almudena Moreno-Borrallo, Sukanya Jana, Shubhrima Ghosh, Eduardo Ruiz-Hernandez

**Affiliations:** † School of Pharmacy and Pharmaceutical Sciences, Trinity College Dublin, the University of Dublin, College Green, Dublin 2 D02 PN40, Ireland; ‡ Trinity Translational Medicine Institute, Trinity College Dublin, the University of Dublin, St. James’s Hospital, Dublin 8 D08 NHY1, Ireland; § School of Biological, Health and Sports Sciences, Technological University Dublin, Grangegorman Lower, Dublin 7 D07 ADY7, Ireland

**Keywords:** Cancer angiogenesis, antiangiogenic factors, pro-angiogenic factors, nanoformulations

## Abstract

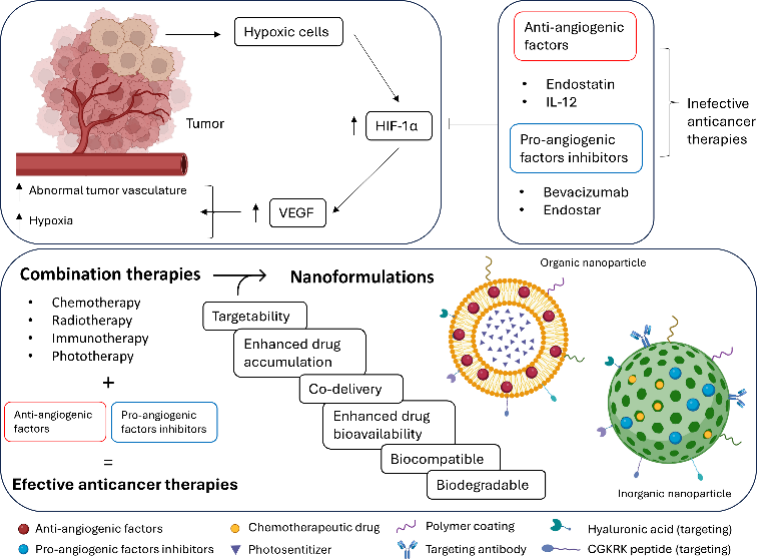

Antiangiogenic medications for cancer treatment have generally failed in showing substantial benefits in terms of prolonging life on their own; their effects are noticeable only when combined with chemotherapy. Moreover, treatments based on prolonged antiangiogenics administration have demonstrated to be ineffective in stopping tumor progression. In this scenario, nanotherapeutics can address certain issues linked to existing antiangiogenic treatments. More specifically, they can provide the ability to target the tumor’s blood vessels to enhance drug accumulation and manage release, ultimately decreasing undesired side effects. Additionally, they enable the administration of multiple angiogenesis inhibitors at the same time as chemotherapy. Key reports in this field include the design of polymeric nanoparticles, inorganic nanoparticles, vesicles, and hydrogels for loading antiangiogenic substances like endostatin and interleukin-12. Furthermore, nanoformulations have been proposed to efficiently control relevant pro-angiogenic pathways such as VEGF, Tie2/Angiopoietin-1, HIF-1α/HIF-2α, and TGF-β, providing powerful approaches to block tumor growth and metastasis. In this article, we outline a selection of nanoformulations for antiangiogenic treatments for cancer that have been developed in the past ten years.

## 1 Introduction

**Figure 1:**
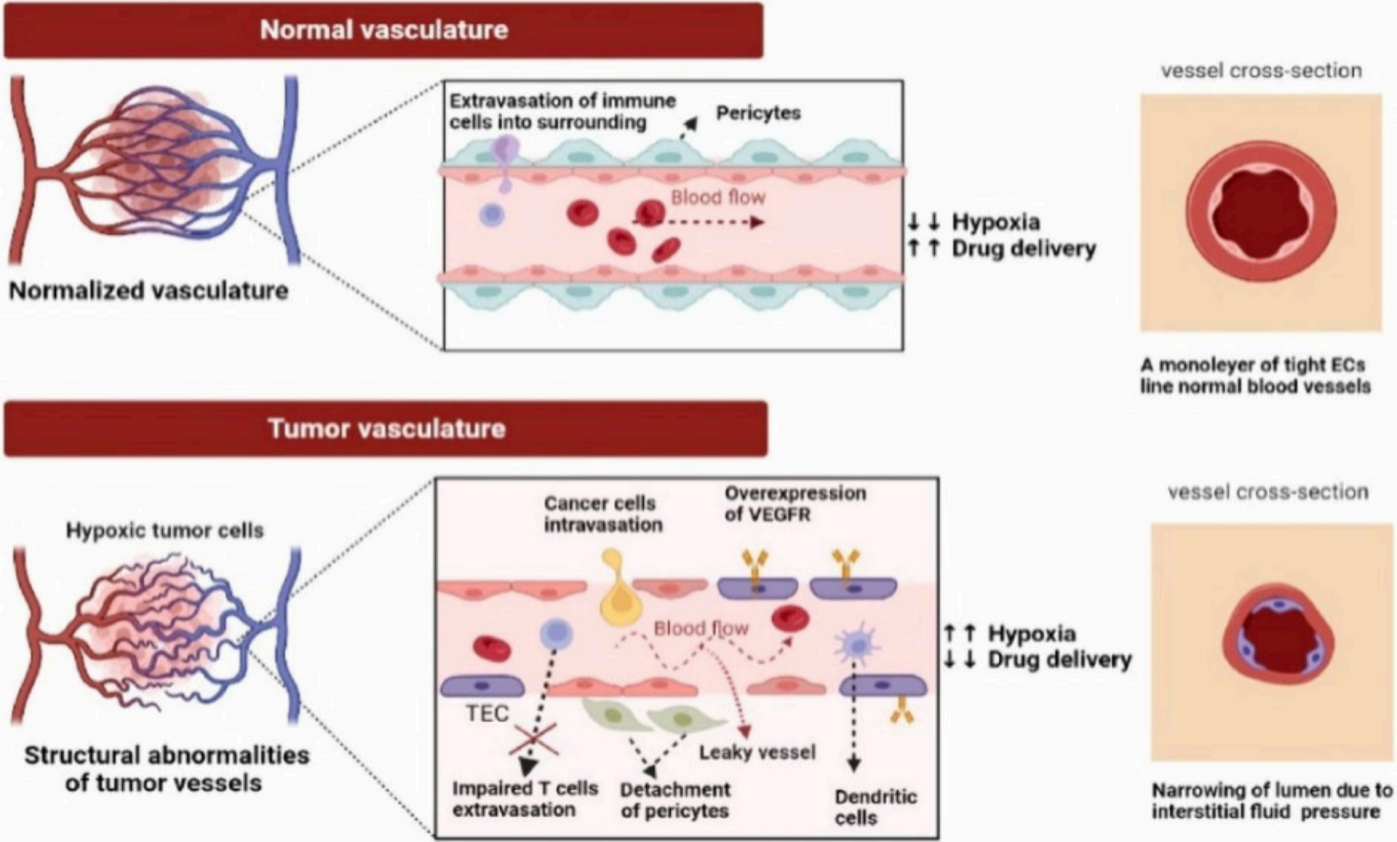
Comparison of tumor and normal vasculature. Unlike normal vasculature, tumor blood vessels lack a mature hierarchical structure, display a discontinuous EC lining, and have inadequate pericyte coverage, leading to vessel leakiness. These structural irregularities result in increased interstitial pressure, which constricts the lumen and hinders the delivery of oxygen and therapeutic drug to solid tumor cells. Tumor-associated ECs, characterized by elevated VEGF receptor expression, replace their normal counterparts, making these vessels more responsive to VEGF. Additionally, reduced T cell extravasation impairs antitumor immune response. Reproduced from ref (9). Available under CC-BY 4.0. Copyright 2023 Elsevier B.V.

For over five decades, researchers have recognized the link between tumor growth and neovascularization, first proposed as a therapeutic target by Folkman in 1971 through his pioneering work on antiangiogenesis therapies.^1^ Angiogenesis, the formation of new blood vessels from existing ones, is critical to tissue growth and becomes essential for tumor expansion beyond 2–3 mm^3^.^2^ In cancer, the process of angiogenesis is typically activated through an imbalance of growth factors that favors pro-angiogenic signals, initiating an “angiogenic switch.” Hypoxia, or oxygen deprivation is one of the primary drivers of this switch. When tumor cells are located over 70–150 μm from blood vessels, the resulting hypoxic environment triggers the expression of hypoxia-inducible factor 1-alpha (HIF-1α)^3,4^ (Figure 1). This factor, expressed by both cancer and stromal cells, plays a significant role in promoting angiogenesis by upregulating vascular endothelial growth factor (VEGF), a key pro-angiogenic mediator.^5,6^

VEGF secretion stimulates nearby endothelial cells (ECs), leading them to proliferate, migrate, and create new, albeit often abnormal, blood vessels due to the uncoordinated nature of angiogenesis in tumors. These vessels are frequently disorganized, highly permeable, and lack structural support, resulting in a hypoxic and acidic tumor microenvironment (TME) that hinders immune cell infiltration, reduces radiotherapy efficacy, and limits drug delivery, ultimately favoring more aggressive cancer phenotypes.^7,8^

### 1.1 Main Cancer Angiogenesis-Related Factors

Undoubtedly, the main factor involved in the regulation of vascular development is the secreted homodimeric glycoprotein VEGF, or rather VEGF-A, a member of the VEGFs family. Five different proteins constitute this group: VEGF-A, B, C, D, and the placental growth factor PLGF, which recognize three tyrosine kinase receptors: VEGFR1 (VEGF-A, B and PLGF), VEGFR2 (VEGF-A) and VEGFR3 (VEGF-C, D). The ligand-induced dimerization of VEGFR1, and especially of VEGFR2, expressed by the vascular ECs initiates the signaling pathway that regulates ECs’ proliferation and migration. Apart from the direct induction of angiogenesis, other VEGF effects on ECs are antiapoptotic signals, necessary to maintain the viability of the immature vasculature, an increased permeability, chemotaxis and the expression of activators and enzymes, that overall allow ECs to grow, produce protein extravasation to remodel their microenvironment, migrate and survive.^9^ In many studies, VEGF has been indicated as one of the strongest predictor factors of recurrence or survival.^10^ Another important factor is the bFGF (or FGF-2), that is part of an extended family of polypeptides commonly expressed in malignant cancers and that interact with four different receptors (FGFR1, 2, 3, and 4).^11^ The interaction of bFGF with FGFR1, 2, and 3 leads to tumor cells proliferation and invasion, thanks to its paracrine effect and the induction of several matrix metalloproteinases (MMPs) and other key proteins.^12^ Furthermore, bFGF seems to stimulate VEGF and to exert with it a synergistic effect *in vivo*.^11^

Among the other pro-angiogenic factors, transforming growth factor (TGF), which consists of two peptides TGF-α and TGF-β, also stimulates ECs growth. Especially TGF-β, once dysregulated in the late stages of the tumor, acts promoting cancer progression, angiogenesis and metastasis.^5,13^ It seems that TGF-β, and particularly its isoform TGF-β1, exerts its pro-angiogenetic effect by both ECs direct activation (induction of metalloproteases) and VEGF stimulation.^10,14,15^

Hypoxia-inducible factors (HIF) constitute a three-member family of heterodimers composed by an oxygen-sensible subunit (HIF-1α, HIF-2α, or HIF-3α) and an oxygen-insensitive subunit (HIF-1β).^16^ The three α subunits are homologous, and all of them can heterodimerize with HIF-1β. Nevertheless, their expression pattern varies in different tissues and stages.^16^ HIF-1α is the best-known factor of this family, and its expression is regulated depending on the presence of normoxic/hypoxic conditions or genetic alterations.^17^ HIF-1α accomplishes its role of mediator of transcriptional adaptative responses to hypoxia, activating several genes involved in oxygen delivery or metabolism, including VEGF, by binding specific sequences named hypoxia-responsive elements (HREs) and thus regulating highly intricated and interconnected pathways. Although HIF-1α expression is enhanced in the majority of human cancers, its correlation with patient mortality or survival varies depending on the type of tumor.^18^

The angiopoietin signaling system has been identified more recently as the second essential angiogenesis pathway after VEGF/VEGFR.^19^ Such a system primarily includes the glycoproteins Ang1 and Ang2 and the tyrosine kinase receptor 2 (Tie2). Ang1 and Ang2 have different expression patterns and exert opposite effects on Tie2. Indeed, while Ang1 is constitutively expressed in perivascular cells and produces endothelial maturation and stabilization, Ang2 is mainly expressed in ECs, especially in the presence of hypoxia and inflammation, and induces vascular permeability, destabilization, and remodelling, contributing to VEGF-dependent angiogenesis.

On the other side, endostatin is one of the strongest endogenous inhibitors of angiogenesis. It derives from type XVIII collagen in epithelial and vascular basement membranes, and it has a broad target among angiogenesis regulatory genes and receptors.^20^ Endostatin has an important role in the inhibition of several MMPs, in blocking VEGF interactions with its receptors by binding VEGFR1, VEGFR2, and VEGFR3, and in the inhibition of cells migration by binding several surface integrins, among other functions.^20^ The importance of this factor, indeed, can only be understood by considering that around 12% of all human genes are modulated by endostatin in human ECs.^21^

Interleukin-12 (IL-12), an essential pro-inflammatory cytokine involved in cell-mediated immunity, has shown a significative antitumor effect in several animal models, probably due to its impact on both innate and adaptative immunity.^22^ At the same time, IL-12 also has a relevant role in the inhibition of angiogenesis through the induction of interferon-γ (IFN-γ).^23^ IFN-γ exhibits angiostatic effects through multiple experimental settings: it minimizes the length and thickness of the angiogenic sprouts *in vitro*, inhibits vessel outgrowth from mouse embryo metatarsal *ex vivo* and supresses colon vessel proliferation *in vivo*.^24^

### 1.2 Standard Treatments

Due to its essential role in the development of cancer angiogenesis, VEGF is the main target of antiangiogenic treatments. Bevacizumab is a humanized anti-VEGF monoclonal antibody that was approved by the Food and Drug Administration (FDA) in 2004 for the treatment of metastatic colorectal cancer, and it is the first drug developed specifically as an antiangiogenic agent.^25^ Bevacizumab, in combination with fluorouracil-based chemotherapy, showed to significantly increase patients’ progression-free survival and has been utilized ever since.^26,27^ A few years later, other drugs targeted to VEGFRs, the tyrosine kinase inhibitors sorafenib (2005), sunitinib (2006), pazopanib (2009), and vandetanib (2011), were approved for the treatment of different malignancies.^28,29^

On the other hand, drugs based on endogenous angiogenesis inhibitors have also been developed, for example Endostar, a human recombinant endostatin approved by FDA in 2005 as a multitarget vascular endothelial inhibitor for the treatment of nonsmall cell lung cancer.^30^ Nevertheless, antiangiogenics do not seem to provide considerable benefits in terms of long-term survival when administered alone, but only in combination with chemotherapeutics.^31^ Thanks to the tumor vasculature normalization produced by the angiogenesis inhibitors, which reduce the general tumor vascular density, thus restoring the normal functionality of the remaining vessels, a more regular delivery of oxygen and drugs to cancer cells is produced with respect to the typically impaired tumor vasculature.^31^ However, optimizing a schedule for the administration of angiogenesis inhibitors and chemotherapeutics, with the aim to avoid an excessive vascular regression or the related side effects on normal vasculature, results challenging. Such side effects normally include, for the forementioned drugs, venous thromboembolism, hypertension, proteinuria, epistaxis, fatigue, and skin toxicity, among others.^32^ Furthermore, it is now clear that prolonged antiangiogenic therapy, especially if monotargeted, does not stop the tumor progression, probably due to a sort of resistance developed by VEGFRs-expressing cancer cells that leads to the activation of multifactorial compensatory pathways, that induce the expression of other pro-angiogenic factors and cytokines able to enhance tumor survival, aggressiveness and metastatic abilities.^31,33^

In this context, nanotherapeutics could help in overcoming some of the issues related with current antiangiogenic treatments.^34^ More specifically, they can precisely target the tumor vasculature providing high tumor accumulation and on-demand drug release, thus reducing undesired side effects, and can also easily allow the contemporary delivery of different angiogenesis inhibitors, possibly combined with chemotherapeutics.^35^ In this review, we summarize a selection of nanotherapeutic formulations for antiangiogenic treatments for cancer developed over the past decade. The studies covered are examples, and the review is not comprehensive of the literature in this field.

## 2 Nanotherapeutic Formulations for Drug Delivery

**Figure 2:**
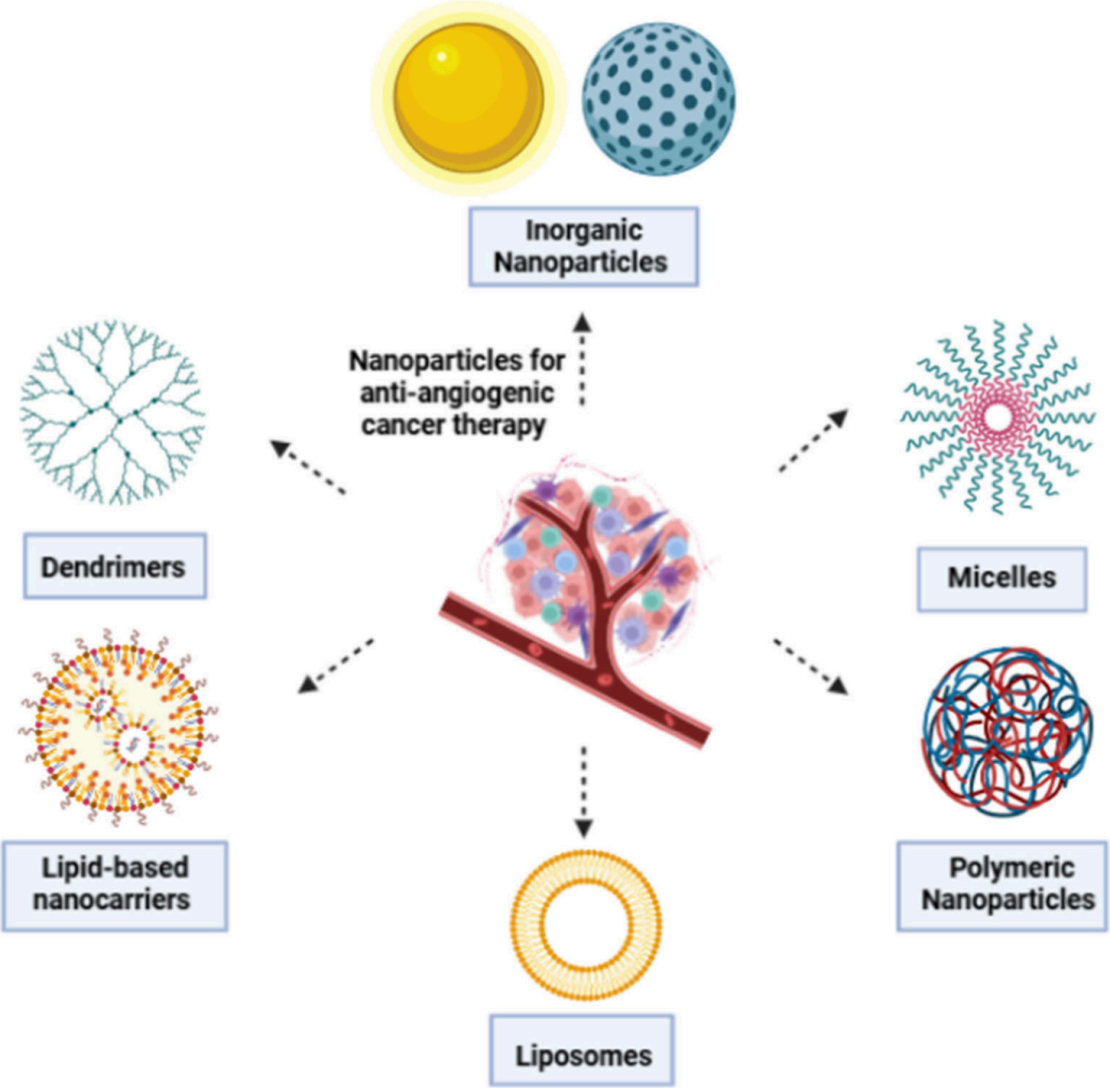
Common NPs used in antiangiogenic therapy for cancer. The Figure illustrates different nanocarriers, including inorganic NPs (e.g., gold, silver, iron oxide), lipid-based carriers (e.g., solid lipid NPs, nanostructured lipid carriers), polymeric NPs (e.g., PLGA, chitosan), liposomes, dendrimers, and micelles.

Various nanocarriers have been developed for the delivery of antiangiogenic agents, each with distinct properties that influence their effectiveness, biocompatibility, and practical applications.^36,37^ These platforms include inorganic nanoparticles (NPs), lipid-based carriers, liposomes and polymeric NPs, micelles, and dendrimers (Figure 2). Their suitability depends on a balance of stability, targeting ability, biodegradability, and drug-loading efficiency.

Inorganic NPs, such as gold, silver, and iron oxide nanoparticles, are distinguished by their stability and unique optical, magnetic, and catalytic properties. Gold NPs (AuNPs), for instance, suppress angiogenesis by generating reactive oxygen species (ROS) upon light exposure, damaging endothelial cells and restricting vascular expansion.^38^ They also enable photothermal therapy, producing heat upon stimulation, to disrupt tumor vasculature. Iron oxide NPs serve both imaging and therapeutic roles, functioning as MRI contrast agents that enable real-time nanoparticle tracking while simultaneously delivering antiangiogenic drugs directly to tumor sites. When conjugated with antiangiogenic drugs, they can be targeted particularly to the tumor vasculature, increasing localized drug concentration.^39^ These NPs offer high stability and can be engineered for targeted delivery, making them valuable in imaging and combination therapy.^40^ However, their nonbiodegradable nature raises concerns about long-term accumulation in organs such as the liver and spleen, potentially leading to toxicity.^41^ To reduce side effects while preserving efficacy, dosage must be precisely controlled.^42,43^

Lipid-based nanocarriers, such as solid lipid nanoparticles (SLNs) and nanostructured lipid carriers (NLCs), have been proposed for delivering antiangiogenic drugs due to their biocompatibility and structural similarity to biological membranes.^44^ These carriers are typically made from biocompatible and biodegradable lipids such as phospholipids, cholesterol, and natural oils, which are well-tolerated by biological systems. The use of renewable, natural sources for these lipids, such as plant and animal oils, makes lipid-based nanocarriers more eco-friendly compared to synthetic alternatives. Furthermore, these nanocarriers can be designed to encapsulate both hydrophilic and hydrophobic drugs, offering versatility in drug delivery applications, including those for antiangiogenic therapy. While the eco-friendliness of lipid-based nanocarriers can be influenced by their synthesis methods, there has been a growing emphasis on using greener production techniques, such as solvent-free methods, and exploring sustainable lipid sources, further enhancing their environmental sustainability. SLNs are formed by solid lipids at room and body temperatures, providing a stable matrix for drug encapsulation. They have the capability to encapsulate both hydrophobic and hydrophilic drugs, making them suitable carriers for antiangiogenic therapies such as bevacizumab. NLCs are an improved variant of SLNs comprising a mixture of solid and liquid lipids. Their structure has a higher drug-loading capacity and increased stability, allowing therapeutic drug levels to be maintained for longer periods of time. Advantages of lipid-based carriers include suitable biocompatibility and low immunogenicity. They can encapsulate a wide spectrum of drugs, while protecting them from premature degradation. Furthermore, lipid nanocarriers improve the bioavailability of poorly soluble drugs and allow targeted delivery via surface changes, which is advantageous for targeting the tumor vasculature.^45^ However, these carriers may present limited drug loading capacities and are susceptible to drug leakage. Stability can in some instances be an issue, as lipids can oxidize and alter drug release characteristics. Also, they can be rapidly removed from the circulation unless surface changes are employed to extend systemic circulation.^44^

Liposomes are spherical vesicles composed of a lipid bilayer that closely resembles cellular membranes, making them highly adaptable to biological systems. These eco-friendly carriers are often derived from natural or renewable lipid sources, reducing their environmental impact. Liposomes can encapsulate both hydrophilic and hydrophobic drugs within their aqueous core and lipid bilayer, respectively. Conventional liposomes are commonly used to encapsulate antiangiogenic drugs such as paclitaxel and doxorubicin. They can be engineered to enhance drug retention and release at acidic tumor sites, allowing for more selective targeting of the tumor vasculature. PEGylated liposomes, in particular, have the added benefit of evading rapid reticuloendothelial system (RES) clearance, resulting in extended circulation times that promote tumor accumulation.^46^ However, liposomes often exhibit stability issues, notably in terms of drug leakage and storage. They are susceptible to oxidation, which may undermine their structural integrity.^47^ Additionally, without PEGylation, they are rapidly eliminated from circulation, reducing their efficacy.^46^

Polymeric NPs are another potential platform for antiangiogenic therapy. Polymers such as poly(lactic-co-glycolic acid) (PLGA), polycaprolactone, and chitosan can be engineered to achieve controlled release and surface functionalization, making them ideal for targeted drug delivery. These biocompatible, biodegradable, and sustainable polymers are derived from renewable resources, minimizing their environmental impact. They can be tailored to encapsulate therapeutic agents, ensuring sustained release at specific sites, and their surfaces can be functionalized for enhanced targeting. The use of these eco-friendly materials contributes to more sustainable drug delivery systems while maintaining high therapeutic efficacy.^48^ PLGA is a biodegradable polymer that breaks down into lactic and glycolic acids and is naturally metabolized in the body. Antiangiogenic drugs encapsulated in PLGA NPs have exhibited a sustained release profile, demonstrating a consistent therapeutic concentration at the target location. These NPs can be loaded with targeted ligands like folate or RGD peptides to improve delivery to tumor blood vessels.^49^ Chitosan is a naturally occurring polysaccharide known for its biocompatibility and mucosal surface adherence, and chitosan-based particles can incorporate antiangiogenic drugs such as small interfering ribonucleic acid (siRNA) targeting VEGF, enabling gene therapy to supress vascular expansion.^50^ Advantages of polymeric NPs include controlled and prolonged drug release, improving therapeutic efficacy and reducing dose frequency. Furthermore, polymeric NPs are also biodegradable, thus reducing the concerns of long-term toxicity.^48^ However, these NPs can be difficult to develop, as their particle sizes and drug loading effectiveness is variable. Degradation rates of these particles may also be inconsistent resulting in unpredictable drug release patterns.^51^ Certain polymers can also elicit immunological responses, necessitating careful formulation and surface modification.^52^

Polymeric micelles are made of amphiphilic molecules with a hydrophilic shell and hydrophobic core that self-assemble at the nanoscale.^53^ They are especially effective in delivering hydrophobic drugs, because they solubilize them in their core. Micelles can be considered eco-friendly when made from natural or biodegradable surfactants, such as those derived from renewable resources such as plant-based lipids, sugars, or proteins. This makes them more environmentally sustainable compared to synthetic petroleum-based alternatives. Additionally, micelles offer an excellent platform for controlled and targeted drug delivery, including encapsulating antiangiogenic factors, which can improve therapeutic outcomes while contributing to more sustainable nanomedicine practices. The overall eco-friendliness of micelles depends on the materials used and the green synthesis methods employed during their production. Hydrophobic antiangiogenic drugs like sunitinib and sorafenib can be solubilized and made bioavailable by micelles based on amphiphilic block copolymers, such as poly(ethylene glycol)-b-(caprolactone) (PEG-b-PCL). They can efficiently accumulate in tumor tissue, because of their small size and long circulation periods. Micelles, in general, facilitate increasing levels of hydrophobic drug absorption and duration in circulation.^54^ Their enhanced circulatory stability and ability to be modified for targeted delivery allow them to accumulate selectively in tumor tissues. However, micelles may not be stable under physiological conditions, which could cause rapid release of drug.^55^ Their structure may break down under specific physiological conditions or at low drug concentrations, decreasing the efficacy of treatment.^56,57^

On the other hand, polymeric dendrimers are highly branched, tree-like macromolecules with numerous attachment sites for drugs, imaging agents, and targeting ligands. Their unique design allows for accurate control over medication loading and release.^58^ Poly(amidoamine) (PAMAM) dendrimers are widely employed in antiangiogenic applications because of their well-defined structure and capacity to encapsulate therapeutic ingredients. They can be functionalized to transport various antiangiogenic drugs, which improves their effectiveness.^59^ Dendrimers offer high levels of control over drug release, allowing for long-term delivery to target areas. Their multifunctional structure enables the codelivery of drugs and imaging agents, hence supporting theranostic applications. They also have a high drug loading capacity due to their branching nature. Nevertheless, dendrimers can be harmful, particularly at higher generations, due to their cationic charges.^60^ Moreover, scalability and extensive use in clinical applications are restricted by the complexity and cost of their synthesis.^61,62^

Each of the above nanocarrier systems presents trade-offs among efficacy, stability, toxicity, and scalability. Inorganic NPs excel in stability and multifunctionality but pose biocompatibility concerns. Lipid-based carriers and liposomes offer superior biocompatibility but suffer from stability and clearance limitations. Polymeric NPs enable controlled drug release but require precise formulation to ensure a consistent performance. Polymeric micelles provide excellent solubilization of hydrophobic drugs but face challenges in maintaining structural integrity, while polymeric dendrimers allow for high drug loading and targeted delivery, but remain limited by toxicity concerns and production costs. The selection of an optimal nanocarrier depends on the specific therapeutic goals, balancing drug delivery efficiency with safety and practicality in clinical applications.

## 3 Antiangiogenic factors and nanotherapeutic agents

Antiangiogenic factors inhibit the growth of new blood vessels in cancer cells; thus, enhancement or up-regulation of their expression levels is an area of cancer therapy research. A major part of the studies related to anticancer therapies using NPs has focused on the delivery of antiangiogenic factors, especially against pro-angiogenic genes such as VEGFR2.^29^ Relevant antiangiogenic factors explored for cancer include thrombospondins, Angiostatin, endostatin, and IL-12, as discussed below.

### 3.1 Endostatin

Endostatin is one of the well-known antiangiogenic factors released by the proteolytic cleavage of collagen XVIII and is reported to be involved in the suppression of neoangiogenesis.^63^ It is also known to downregulate many signaling cascades involving coagulation and adhesion as well as (tumor necrosis factor alpha) TNF-α, nuclear factor kappa B (NFκB), and ephrin expression. Endostatin has been explored as an anticancer drug in clinical trials. Moreover, endostatin enhances oxygen delivery while also improving the effectiveness of radiotherapy.^64^

One recent study involved delivery of the secretory endostatin gene (pVAXI-en) loaded onto a tandem peptide TAT (Transactivating Transcriptional Activator)-AT7-modified PEI (polyethylenimine) with heterobifunctional PEG linker into U87 glioma cells *in vitro* and *in vivo*. ATWLPPR (AT7) (identified by a phage peptide library) showed high affinity to VEGFR2 and NRP-1 at the same time. The aim was to evaluate its binding affinity to VEGFR-2 and NRP-1, vasculature-targeting ability, and blood brain barrier (BBB) crossing capacity. Both VEGFR2 and NRP-1 are overexpressed in vasculature endothelial or glioma cells and have a synergistic effect on angiogenesis. The above formulation was effective in inhibiting VEGFR2 and NRP-1, significantly suppressing the tube formation and migration of ECs, inhibited glioma growth, and reduced the microvasculature in orthotopic U87 glioma-bearing nude mice.^65^ Similarly, another study by Adeyemi et al. used a LyP-1 homing peptide as a targeting agent for the p32 receptor on the outer layer of a polymeric PEI-PEG-chitosan-grafted nanosystem containing endostatin. In this case, the formulation was designed for delivering endostatin intracellularly and targeting mitochondria in a squamous cell carcinoma cell line (KYSE-30) model. The VEGF-C and MMP-2 angiogenic factors were studied by observing the nanosystem effects on endostatin release and tumor necrosis in nude mice with a KYSE-30 cell xenograft. Using LyP-1 (a homing peptide for p32 receptor targeting) to guide NPs carrying endostatin can effectively deliver the drug to specific sites to achieve strong antiangiogenic effects in squamous cell carcinoma. The LyP-1-modified nanosystem containing endostatin showed improved effectiveness in fighting against KYSE-30 cells by directly attacking tumor lymphatics, causing nucleus rupture, degrading mitochondria, reducing cell growth and movement, and inhibiting VEGF-C and MMP2 expression. A significant decrease in tumor size was observed in mice after treatment: 43.25% with the control, 41.36% with the NPs, and 61.01% with the LyP-1-modified nanosystem. Conjugating a homing peptide like LyP-1 onto the endostatin-loaded nanosystem significantly improved endostatin release and increased *in vitro* and *in vivo* antitumor effectiveness for squamous cell carcinoma compared to the nontargeted nanosystem.^66^

The research by Wu et al. focused on the formulation of microspheres for lung-targeted delivery and the sustained release of recombinant human endostatin (rhES). The targeting effectiveness was assessed through *in vivo* imaging with quantum dots (QDs). Amphiphilic polymers [poly(ethyl acrylate-co-butyl methacrylate-co-methacrylic acid)] were used to coat oil-soluble QDs in order to create a polymer-quantum dots micelle (QDs-M) that could remain stably dispersed in water. rhES-QDs-M microspheres (rhES-QDs-M-MS) were obtained by using electrostatic spray technology and tested *in vivo*. The microspheres, with a size of 4–8 μm, remained stable in water and displayed excellent optical characteristics. Moreover, they showed a continuous rhES release that lasted for a minimum of 15 days (with over 80% released) without any sudden or rapid release. The rhES-QDs-M-MS demonstrated a strong safety profile and significantly suppressed the growth of human umbilical vein ECs (HUVECs) by approximately 70%. The pharmacokinetic data in ICR mice indicated the presence of rhES even after 72 h, highlighting the considerable sustained-release impact of rhES-QDs-M-MS. The microspheres also showed improved lung targeting and increased antitumor effects in comparison to the rhES alone. The trackable rhES-QDs-M-MS acted as a potential drug delivery method for the unstable rhES protein, enhancing lung-targeted impact, sustained-release capabilities, and anticancer effects.^67^

In another study by Rezaei et al., the authors showed that the N-terminal segment of endostatin, encompassing residues 1–27, effectively replicated its antiangiogenic and antitumor properties. The mutant N-terminal peptide, known as the ES-SS peptide, in which the Zn-binding loop is substituted with a disulfide loop, retains its antiangiogenic and antitumor properties similar to those of the native peptide.^68^ For a general comparison, different liposome-peptide molar ratios of phosphocholine to improve the durability and half-life in the bloodstream were considered. The results from the release experiments indicated that the liposomal peptide was fully released within a week. An increase of cell survival, with an IC_50_ value of around 0.1 μM, when compared to the peptide without encapsulation (0.07 μM), was demonstrated. Additionally, the authors report that coarse-grained molecular dynamics simulation showed peptides gathering in the hydrophilic core of the liposome, which helped stabilize its structure. A recombinant human endostatin was also loaded into carboxymethyl chitosan NPs, as reported by Xia et al. and subsequently embedded in a 3D hydrogel. After peritumoral injection, the NPs were released, and they invaded the solid tumor, cross-linking with intratumoral calcium ions. This cross-linking process enabled the formation of larger particles and, therefore, leading to longer retention in tumor.^69^

Due to its short half-life, endostatin is primarily given systemically, resulting in limited delivery to tumor tissue and frequent toxic systemic side effects. Hyaluronic acid-tyramine (HA-Tyr) was used to produce a locally injectable hydrogel loaded with endostatin. It showed a higher ability to impede the growth of HUVECs in the MTT assay. Moreover, it demonstrated a more effective impact on HUVEC invasion and a stronger antiangiogenic effect, leading to reduced overall toxicity in Lewis lung cancer (LLC)-bearing mice.^70^

Another study involved combinatorial therapy with the endostatin gene transfected into tumor-migrating decidua mesenchymal stromal cells (DMSC) via nucleofection. Additionally, doxorubicin-loaded mesoporous silica NPs were introduced into DMSC, which acted as the drug vehicle. This final platform was found to provide an effective strategy for migration to tumors and apoptosis, as tested in monolayer NMU cancer cells and NMU-HUVEC 3D-spheroid cell cultures, due to the combinatorial chemotherapeutic effect of doxorubicin and the antiangiogenic effect of endostatin gene therapy.^71^

### 3.2 Interleukin-12 (IL-12)

IL-12 is a cytokine widely produced by macrophages and B-cells and known for its antiangiogenic and antitumor effects. It induces the production of IFN-γ and inducible-protein 10 (IP-10), both of which are reported to inhibit neovascularisation. It is also known to suppress the aberrant expression of pro-angiogenic factor VEGFR-3.^72^ However, systemic administration of the IL-12 protein is reported to have severe adverse side effects, and IL-12 gene delivery is a safer alternative to the protein delivery.^73^ Thus, the delivery of IL-12 genes provides a potential strategy for inhibiting angiogenesis and proliferation in tumors.

pH-responsive metformin-based-PEG micellar NP systems with doxorubicin and IL-12 plasmid (pIL-12) load have been explored as an antiangiogenic and antiproliferative formulation in 4T1.2 breast cancer cells. A PEG-carboxydimethyl maleate polymer (PEG2K-cdm) and polymetformin (PMet) polymers were combined by ring-opening reaction to form PMet-P(cdmPEG2K), and doxorubicin was loaded by a thin film hydration method. IL-12 was further loaded, based on the ratio of the number of amino groups in PMet-P(cdmPEG2K) to the number of phosphate groups in pIL-12. The PEG layer was found to have a deshielding effect which led to more plasmid DNA being delivered into the tumor cells and subsequently increased IL-12 expression. A cell-death rate of 66.5% was observed in doxorubicin /pIL-12 loaded particles *in vitro*.^74^ In a similar study, a self-assembled metformin-based nanosystem with physically encapsulated doxorubicin, complexed with IL-12 plasmid load and functionalized with hyaluronidase-responsive thiolated hyaluronic acid, was delivered to 4T1 breast cancer cells.^75^ It had a longer presence in the bloodstream, gathered effectively in tumors, and was taken up by tumor cells through CD44 receptor-targeted tumor-specific delivery. Subsequently, corelease of doxorubicin/pIL-12 occurred in the endo/lysosomes of the TME. Furthermore, this nanosystem showed superb transfection of pIL-12 and expression of IL-12 in tumors of mice with 4T1 tumors. They cooperatively boosted NK cells and cytotoxic T lymphocytes within tumor infiltrates while shifting anti-inflammatory M2 macrophages toward pro-inflammatory M1 macrophages, reducing Treg cells. This led to higher levels of IL-12, IFN-γ, and TNF-α cytokines, resulting in enhanced antitumor and antimetastatic effects in a 4T1 breast cancer lung metastasis mouse model. As an alternative route, nonviral delivery of IL-12 into tumor associated macrophages (TAMs), which inherently secrete IL-12, as well as into cancer cells was studied using the esterase-responsive polymer, poly[N-[2-(acryloyloxy)ethyl]-N-[p-acetyloxyphenyl]-diethylammonium chloride], which formed a polyplex with IL-12 plasmid. Further coating with PEG and conjugation to aminoethyl anisamide, a ligand targeting sigma 1 receptors, was undertaken, and their effects were studied in Raw 264.7 and KPC cells. The above gene delivery system was found to enhance IL-12 production in TAMs, changing the TME by converting them from M2 to M1 type while inducing anticancer immune responses.^76^ PAMAM forms one of the widely explored nonviral delivery vectors for IL-12. Modified cholesterol-grafted PAMAM-alkyl-PEG was used to deliver the plasmid encoding IL-12 to colon cancer cells. These modified PAMAM dendrimers demonstrated a higher level (increased by approximately 2-fold) of IL-12 expression when compared with the unmodified nanosystems. The enhanced hydrophobic/hydrophilic balance using both PEG and cholesterol modification are thought to be responsible for the elevated gene transfer and expression of IL-12.^77^

Another study showed that IL-12-loaded poly(lactic-co-glycolic acid) (PLGA) nanospheres can enhance treatment effectiveness and prevent negative side effects seen in past human trials. Studying the immune responses in healthy BALB/c mice suggested the protective effects of IL-12, as the nanospheres boosted pro-inflammatory cytokines/chemokines in the blood and tissues without causing harmful changes in immune cell genes. Gene expression profiling showed that pro-inflammatory signaling pathways were activated in systemic tissues, which is probably where these effector cytokines originated. These data confirmed that the dynamics of nanospheres, such as protecting IL-12 from immune cells in the bloodstream, distributing to immune tissues in the body, and gradually releasing active cytokines, are crucial for a safe immunostimulatory treatment.^78^ PLGA nanospheres, which are approved by the FDA for drug delivery, could potentially be used to transport IL-12 to tissues without disrupting normal homeostasis. These IL-12-loaded nanospheres (<1 μm in diameter) could be used for cancer treatment and they could offer future perspectives and advancements in nanoscale tumor immunotherapeutics.^79^ Another study by Sousa et al. revealed that a new PLGA-nanoadjuvant containing immunostimulatory NPs (ISN) with IL-12 could reduce IL-12 side effects and boost the immune response. ISN significantly enhanced the release of inflammatory cytokines in both glioblastoma cancer cells (e.g., IL-8 expression increased by 2.6 times compared to free IL-12) and macrophages (e.g., TNF-alpha expression increased by 2 times and IL-6 expression by 6 times compared to free IL-12). Furthermore, the transport of IL-12 inside cells using ISN was successful, leading to changes in pro-inflammatory cytokine levels at both transcriptional and protein expression levels.^73^

On the other hand, polymeric NPs using polylactic acid copolymers, responsive to the tumor acidic environment, as the core, and Pluronic F127 as a hydrophilic thermo-sponge shell were prepared to achieve the codelivery of the chemotherapeutic drug paclitaxel (PTX) and the immune cytokine IL-12. The synthesized polymeric NPs were accumulated at the tumor location, effectively suppressing the proliferation and spread of breast cancer cells 4T1 and extending the overall survival of mice with tumors. The pairing of PTX and IL-12 triggers T lymphocytes and NK cells to produce IFN-γ, targeted regulatory T cells, and promote the transformation of tumor-associated macrophages into the M1 type, thus enhancing the immune-suppressive conditions within tumors. It was demonstrated that the combination of PTX and IL-12 can eliminate tumor cells and also control the tumor immune environment at the same time.^80^

**Figure 3:**
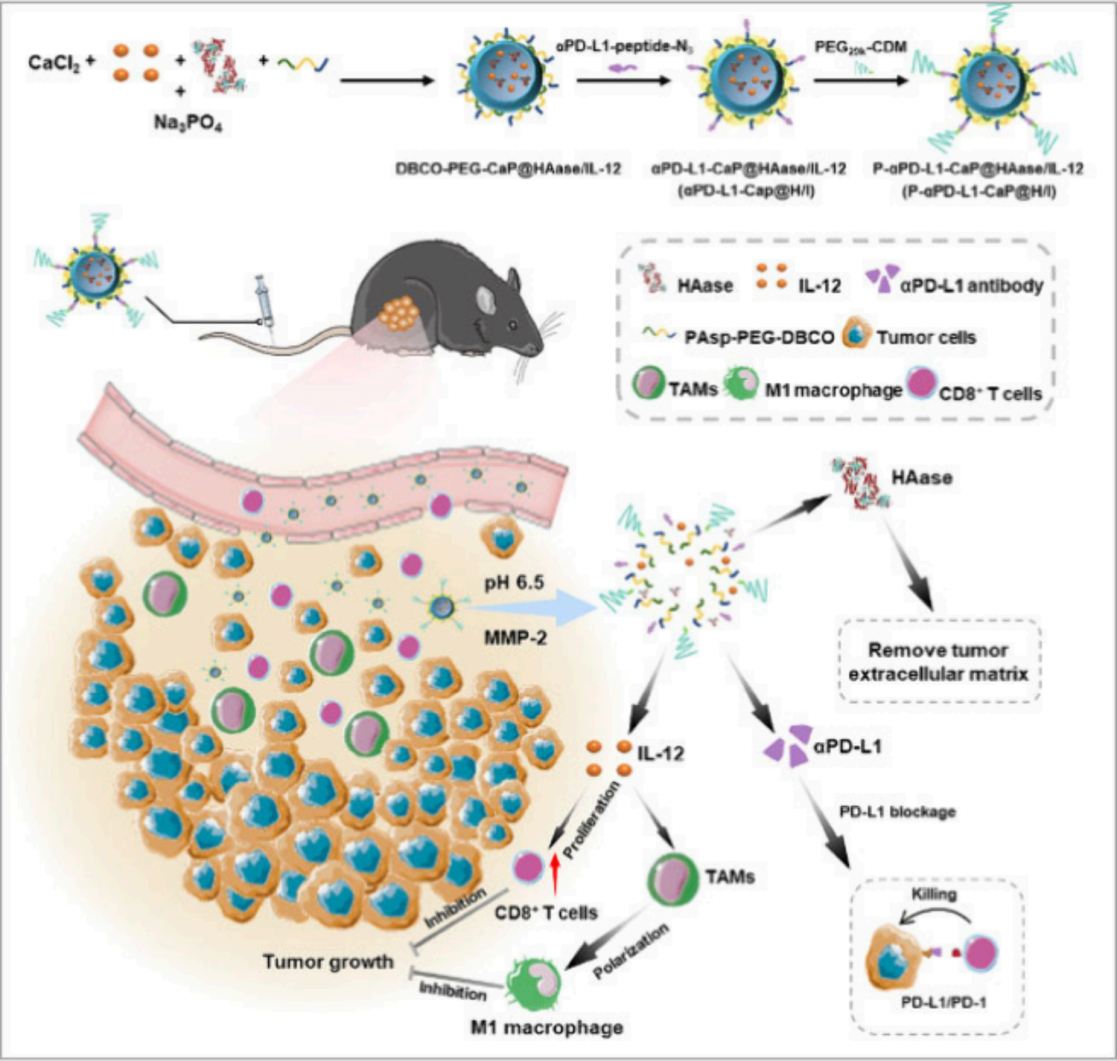
pH and MMP-2 responsive polymer/CaP hybrid nanocarrier for codelivery of HAase, IL-12, and αPD-L1 to enhance antitumor immune response in hepatocellular carcinoma. Reproduced with permission from ref (81). Copyright 2023 Elsevier B.V.

It is well-known that the thick extracellular matrix (ECM) hinders the entry of cytotoxic T lymphocytes (CTLs) into tumors, resulting in limited effectiveness of T-cell-based immunotherapy for hepatocellular carcinoma (HCC). In order to solve this problem, a pH and MMP-2 responsive polymer/calcium phosphate (CaP) hybrid nanocarrier was utilized to simultaneously deliver hyaluronidase (HAase), IL-12, and anti-PD-L1 antibody (αPD-L1) (Figure 3). The tumor acidity-induced dissolution of CaP enabled the release of HAase, which digests ECM, and IL-12 promoted CTL infiltration and proliferation, respectively. The release of αPD-L1 inside the tumor, instead, mitigated the immunosuppression of T cells by tumor cells, preventing them from evading the cytotoxic effects of CTLs. This combination strategy effectively triggered a strong immune response against tumors, stopping the growth of HCC in mice. Moreover, the tumor accumulation of the nanocarrier was improved by an acid-sensitive PEG coating while also decreasing immune-related adverse events caused by αPD-L1. This nanodrug, which is sensitive to two different stimuli, presents a successful immunotherapy approach for solid tumors with a dense extracellular matrix.^81^

Another study developed a gene carrier using N-[(2-hydroxy-3-trimethylammonium)propyl] chitosan salt (HTCS) to deliver a plasmid containing the IL-12 gene. The modification of chitosan through quaternization resulted in increased transfection efficiency of HTCS when compared to unmodified chitosan and PEI. This change resulted in polyplexes with an optimal size of approximately 75 nm, enabling their administration through various routes, such as intravenous and intratumoral injections. Therefore, the formulation of polyplexes using chitosan, a material that is cytocompatible, water-soluble, and cost-effective, with a transfection efficiency surpassing that of PEI, could lead to new possibilities for translating polymeric delivery systems from the lab to clinical use.^82^

A new NP (CaCO_3_-polydopamine-polyethylenimine, CPP) was developed by Shen et al. for efficient IL-12 delivery, with the aim of inhibiting cancer advancement via immunotherapy. CPP can efficiently induce the expression of plasmid pEGFP-N1 or IL-12 and enhance levels of ROS. Moreover, laser treatment (2.0 W/cm^2^, 808 nm, 1.5 weeks, 3 min)-enhanced CPP-IL-12 resulted in a 2-fold increase in the number of dead or apoptotic cells. The novel CPP-IL-12 demonstrated an increase in IL-12 levels in melanoma cells and promoted immunotherapy for melanoma by stimulating immunogenic cell death CD4+ T cells and improving the activity of tumor-targeting CD8+ T cells, demonstrating CPP-IL-12 potential for tumor treatment.^83^ In another research project, Zhao et al. developed a CaCO_3_ NP coated with a cancer cell membrane modified with cyclic arginyl-glycyl-aspartic acid peptide (cRGD) to deliver IL-12 messenger ribonucleic acid (mRNA). These NPs can acquire the ability to cross the blood-brain barrier and target brain tumors effectively. CaCO_3_ NPs loaded with IL-12 mRNA served as the central element, enabling a combined immunotherapy of necroptosis-triggered immune reaction and IL-12 mRNA delivery using ultrasound stimulation.^84^ Curcumin-synthesized Au-NPs were also prepared to deliver the IL-12 gene safely and effectively to cervical cancer cells. The stability and binding ability of the therapeutic gene to Au-NPs were enhanced by the PLL-PEG functionalization. The PEG coating boosted the delivery and absorption of IL-12-DNA, resulting in an advantageous expression in mRNA and protein levels.^85^

Photothermal therapy (PTT) in the NIR-II biowindow has shown promise as a treatment option, but its effectiveness is constrained by uneven heat distribution and lack of control over metastatic lesions. Significant attempts have been made to address the limitations of PTT through the integration of immunotherapy, yet current approaches continue to face challenges such as low response rates, resistance, or severe immune-related side effects.^86^ In a particular study, a new NP (CSP, containing CuS within SiO_2_ pores and PDMAEMA polycation on the SiO_2_ surface) was investigated for targeted tumor PTT using NIR-II light and simultaneous delivery of genes to induce local production of IL-12 cytokine for in situ immunotherapy. The CSP combined with the plasmid containing the IL-12 gene (CSP@IL-12) showed high gene delivery efficacy, remarkable NIR-II PTT performance, and superior therapeutic results in experiments conducted both *in vitro* (using murine melanoma cell line B16F10 and mouse embryonic fibroblast cell line NIH 3T3) and *in vivo* (using male C57BL/mice). Moreover, it increased the proliferation of CD8+ T cells and promoted their infiltration to eliminate potential metastatic lesions through abscopal effects. Therefore, this innovative combination of NIR-II PTT and IL-12 cytokine treatment could offer a more effective, manageable, and safer option for upcoming photoimmunotherapy.^87^

A potential solution to the problem of a weakened immune system from chemotherapy was offered by creating a nanostructure comprising hollow mesoporous silica NPs connected by thioketal bonds and coated with carboxymethyl chitin through electrostatic attraction. Additionally, a glucose-regulated protein 78 binding peptide was added to the surface for loading doxorubicin and α-tocopheryl succinate (α-TOS). The drug-free NPs exhibited excellent biocompatibility with 4T1 cells and RAW264.7 macrophages. The release from this nanostructure can be triggered by pH and H_2_O_2_ levels in the TME to release IL-12, doxorubicin, and α-TOS locally. This combination therapy significantly increased the effectiveness of anticancer treatment, boosted immune responses by switching tumor-associated macrophages to tumoricidal M1 types that reduce the inflammatory response in tumors and encourage angiogenesis and tissue remodelling, and lowered lung metastasis while minimizing side effects. Therefore, the synthetized nanostructure shows potential as a compelling approach for merging chemotherapy and local macrophage modulation-immunotherapy in cancer treatment.^88^

On the other hand, nonviral gene carriers based on PEI show great potential for delivering nucleic acids in the fight against cancer. Yet, their limited degradability often leads to significant cytotoxicity, which in turn greatly impedes further clinical applications.^89^ A collection of biodegradable cationic polycarbamates (CPCs) was created to have chemical compositions comparable to those of PEIs for gene delivery. These CPCs showed changes in degradation patterns under natural conditions and broke down faster when containing secondary amino groups in comparison to primary amino residues. Furthermore, these CPCs exhibited positive gene delivery characteristics such as robust gene condensing ability and high buffering capacity while also showing lower levels of cytotoxicity compared to nondegradable PEI controls. As a result, they led to effective gene transfection in various cell types *in vitro*. Specifically, the secondary amino-containing CPCs using N-methyl-2,2′-diaminodiethylamine (referred to as BHE-MDE) exhibited superior *in vitro* transfection efficiency in type-II alveolar epithelial cells. The BHE-MDE showed similar transfection efficiency in epithelial cells compared to the 25 kDa linear PEI used as a positive control. In a C57BL/6 mouse model, BHE-MDE resulted in significant transgene expression in the lung and noticeable IL-12 expression in the peripheral blood. In this way, the BHE-MDE-induced IL-12 production provided effective gene therapy for metastatic LLC cells in the lungs of mice, leading to an extended survival period.^90^

Another study relates to the formulation of GEN-1, comprising of human IL-12 gene expression plasmid and a synthetic plasmid delivery system, delivered into the intraperitoneal cavity of patients with advanced ovarian cancer to produce local and persistent levels of IL-12. This also induces the production of other immunomodulatory cytokines, such as IFN-γ. GEN-1 consists of a plasmid vector encoding the p35 and p40 subunits of human IL-12 genes under a promoter and a synthetic lipopolymer delivery system composed of PEG-PEI-cholesterol. Cholesterol facilitates cellular uptake, while PEG provides stability *in vivo*, and local delivery ensures diminished systemic toxicity of IL-12. The NPs were found to be resistant to degradation by DNases, and they also provided a sustained release of IL-12 for days in mice with disseminated ovarian cancer. Additionally, a combinatorial therapy of the above NPs with carboplatin and paclitaxel had significantly prolonged survival compared to the control.^91^

Another research effort supported the utilization of gemini cationic lipids (GCLs) along with zwitterionic helper lipid DOPE as nanovectors for delivering plasmid DNA encoding IL-12 (pCMV-IL12) into cells. GCLs performed successful pCMV-IL12 transfection with cytokine levels similar to or higher than those achieved with Lipofectamine 2000, used as a control. In addition, the nanovectors exhibited minimal toxicity and high cell viability. The lipoproteins and serum albumin found on the nanovector surface were predominantly responsible for extending the time of blood circulation, offering valuable benefits.^92^ As an alternative route, another study proposed a dual approach involving stereotactic body radiation therapy (SBRT) and direct administration of IL-12 mRNA lipid NPs to pancreatic murine tumors. This therapy successfully treated both primary and metastatic cases, leading to recoveries in C57BL/6J and B6.129S7-Ifng^tm1Ts^/J(IFNg^–/–^) mice mainly confined to the tumor. Exhausting CD4 and CD8 T cells eliminated the treatment effectiveness, demonstrating their importance in treatment outcomes. Single cell RNA sequencing of tumors treated with SBRT/IL-12 mRNA showed a lack of T cell exhaustion as well as a large presence of highly proliferative and effector T cell subtypes. SBRT induced expansion of T cell receptor clones, while IL-12 activated these cells to perform as effectors.^93^

Using cytokine therapy to stimulate a proinflammatory immune response in immunologically cold tumors like high grade ovarian cancers is appealing, but historically its use has been hindered by significant toxicity.^94^ Layer-by-layer (LbL) NPs consisting of a liposomal NP with IL-12 bound to the liposomal surface and subsequently covered with a bilayer of poly-l-arginine and poly-l-glutamic acid have been proposed. The outer layer bound to cancer cell membranes effectively targeted and activated the adaptive immune system for treating multiple orthotopic ovarian tumor models, including immunologically cold tumors. IL-12 treatment via systemically administered LbL NPs demonstrated decreased severe side effects and retained anticancer effectiveness in comparison to IL-12 without carriers or liposomal NPs without layers, resulting in a 30% full survival rate.^95^ Another study demonstrated that using a triblock compound MPEG_2000_ (methoxy polyethylene glycol, molecular weight 2000 g/mol) -PDLLA_4000_ (poly(D,L-lactic acid), molecular weight 4000 g/mol)-MPEG_2000_ modified by cationic liposome 1,2-dioleoyl-3-trimethylammonium propane (DOTAP) could be used as a nonviral vector DOTAP/MPEG_2000_–PDLLA_4000_–MPEG_2000_ (DMPM) to effectively transfer IL-12 plasmid (pIL-12) into tumor tissue and it showed good biodegradability *in vivo*. After pIL-12 was entrapped by the complex of DOTAP/MPEG_2000_–PDLLA_4000_–MPEG_2000_ (DMPM) *in vitro*, the pIL-12 gene entered the tumor tissue through local administration and successfully expressed IL-12 to play an antitumor role through regulating the immune system. The pIL-12 transferred by DMPM was highly expressed both in CT26 cells and B16-F10 cells. These results confirmed that pIL-12/DMPM therapy significantly reduced tumor growth by 8 times in a mouse model.^96^

HC/pIL-12/PMet (cisplatin-hyaluronic acid-interleukin-12 gene-polymetformin) micelleplexes were created by Sun et al. for tumor targeting, effectively delivering both the chemotherapeutic drug cisplatin (CDDP) and the immunotherapeutic cytokine pIL-12 for chemoimmunotherapy of lung cancer. Combining CDDP and pIL-12 in micelleplexes demonstrated massive cancer cell remission, including cellular shrinkage and nuclear fragmentation, indicating a potent antitumor effect in female ICR and C57 mice with lung cancer. Hence, the joint use of CDDP and pIL-12 could eliminate tumor cells and adjust the tumor immune environment at the same time, potentially leading to improved chemoimmunotherapy.^97^ In a similar study, a new nanosystem based on PMet was developed to deliver both doxorubicin and pIL-12 for the combined treatment of metastatic breast cancer. Cationic PMet formed micelles easily to encapsulate doxorubicin and complex with pIL-12, and then a hyaluronidase-sensitive thiolated hyaluronic acid (HA-SH) was added to create micelleplexes. These micelleplexes containing doxorubicin/pIL-12 showed extended time in blood circulation, effective buildup in tumors, and uptake in tumor cells through CD44 receptor-mediated targeting for tumors. Furthermore, doxorubicin/pIL-12 was simultaneously released in endo- and lysosomes within the TME, triggered by HAase. Additionally, these micelleplexes showed an outstanding transfection of pIL-12 and expression of IL-12 in tumors of mice bearing 4T1 tumors. Crucially, these micelleplexes had a synergistic effect on boosting NK cells and cytotoxic T lymphocytes infiltrating tumors as well as shifting M2 macrophages to M1 macrophages and reducing Treg cells. This was accompanied by increased expression of IL-12, IFN-γ, and TNF-α cytokines, resulting in improved antitumor and antimetastatic activity in the 4T1 breast cancer lung metastasis mouse model.^75^

On the other hand, extracellular vesicles (EVs) are a promising class of nanosized compartments explored for delivery of therapeutics. EVs secreted by MC38 colon cancer cells, which overproduce IL-12 and/or shRNA (small hairpin RNA) targeting transforming growth factor beta (TGF-β1), were applied in the form of sole treatment as well as in combination with dendritic cell-based vaccines with significant cytotoxic effect. Merging stem cell technology with nonviral gene therapy techniques could enhance the effectiveness of delivery systems. Mesenchymal stem cells possess a range of benefits as vehicles such as tumor-targeting potential. Poly(amidoamine) (PAMAM, G5) was presented for the transfer of a plasmid containing IL-12 to MSCs. The findings indicated that alkyl-peptide modified PAMAM, which had low toxicity, demonstrated greater efficacy in transporting green fluorescent protein and IL-12 genes to stem cells compared to PAMAM.^98^

### 3.3 Other Antiangiogenic Factors

Several other antiangiogenic factors have also been explored for their effect in cancers. A study devised coexpressing plasmid bearing DES 12 with chemokine IP10 encapsulated within a DOTAP/cholesterol NP, and was found to act an angiogenesis inhibitor in colon and lung carcinoma.^99^ Self-assembled antiangiogenic plasmid (ANG)-bearing hydroxyapatite NPs (ANG/NHAP) were explored for gene-therapy against breast cancer. Conjugation between the negatively charged ANG and cationic NHAP happened via electrostatic interactions. HAP NPs are widely considered for their biocompatibility, large surface area, and ease of fabrication. ANG/NHAP NPs were found to decrease the expression of pro-angiogenic VEGF in MCF-7 breast cancer cells. However, in this case, cell death and effect on tumor vasculature needed to be studied further.^100^ NP drug carriers codelivering oxygen-generating MnO_2_ and sorafenib have also been explored, which can break down H_2_O_2_ into oxygen, reducing hypoxia-induced drug resistance, thus enhancing antiangiogenic effects and improving treatment outcomes for digestive tumors.^101^ Other instances of antiangiogenic effects have been achieved through codelivering antiangiogenic agents and hypoxia-specific siRNA through lanthanide-integrated supramolecular polymeric NPs.^102^

## 4 Pro-angiogenic Factors and Nanotherapeutic Agents

**Figure 4:**
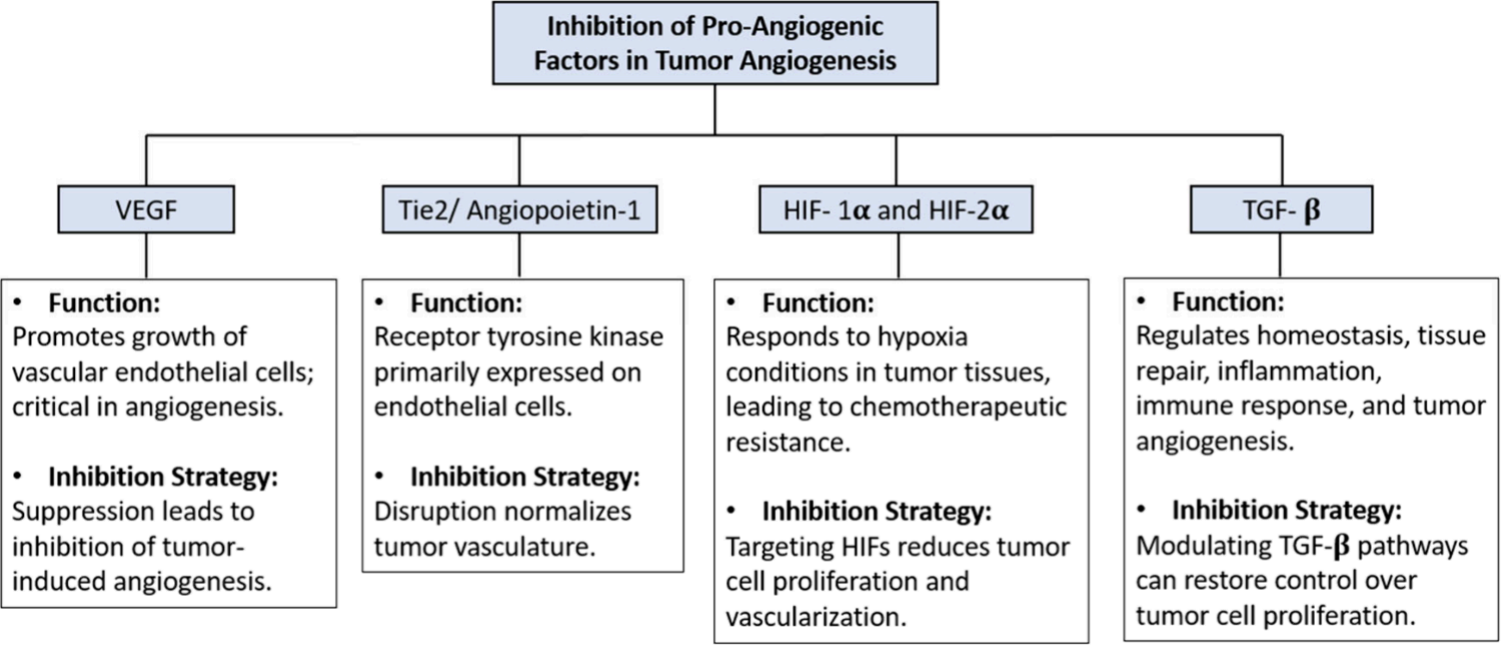
Schematic representation of pro-angiogenic factors involved in tumor angiogenesis.

Delivery systems have been designed to cause the inhibition of relevant factors promoting tumor angiogenesis (Figure 4), some of which are discussed below.

### 4.1 Vascular Endothelial Growth Factor (VEGF)

VEGF is a highly specific growth factor responsible to promote the growth of vascular ECs and plays a crucial role in angiogenesis stimulation. It is often overexpressed in tumor tissues, where it facilitates the survival and growth of cancer cells. Thus, suppression of its expression could result in tumor-induced angiogenesis inhibition, thereby regulating tumor growth and metastasis.^103^

Self-assembling smart NPs were developed using amphiphilic copolymer polyethylenimine-block-polylactic acid (PEI-PLA)/polyethylene glycol-block-poly(l-aspartic acid sodium salt) (PEG-PASp) to load and deliver PTX and VEGF-siRNA and analyze its potential application in breast cancer treatment. When *in vitro* pharmacodynamic studies were conducted to determine gene silencing efficiency, PTX-siRNA-VEGF-NPs successfully silenced VEGF mRNA expression in the 4T1 cell line, while also exhibiting cytotoxicity and inducing apoptosis. *In vivo* 4T1 breast cancer mouse orthotropic model revealed superior anticancer efficacy of PTX-siRNA-VEGF-NPs as compared with both PTX-NPs and siRNA-VEGF-NPs groups. This superiority may be attributed to PTX directly suppressing the tumor and VEGF-siRNA hindering tumor growth by reducing expression of VEGF, thus inhibiting necessary nutrition and blood supply.^104^

In another study by Le et al., poly(β-amino ester) (PBAE) NPs were prepared to deliver bevacizumab-encoded synthetic mRNA as an anti-VEGF antibody therapy for the treatment of nonsmall cell lung cancer (NSCLC). In NSCLC, the main targets of VEGF for promoting angiogenesis are pulmonary ECs (PECs). Thus, delivering mRNA selectively to PECs to produce bevacizumab could effectively block VEGF-driven angiogenesis and hinder tumor progression. To enhance PEC targeting, a range of PBAE polymers and specific PBAE-D (dodecyl amine) variants were evaluated based on endosomal escape behaviors and mRNA transfection efficiency. Results indicated that the transfection site and delivery efficiency were influenced by the content, tail length, and molarity of the polymer formulation. *In vitro* cytotoxicity tests revealed that bevacizumab mRNA loaded PBAE NPs treatment significantly reduced cell viability of HeLa cells. The particles were further tested on primary human umbilical vein ECs, which exhibited pronounced cell viability. Thus, PBAE-based NPs, delivery of PEC-selective bevacizumab mRNA, and VEGF blockade in pulmonary tissues were achieved.^105^

Micelles made of biodegradable polyethylene glycol-poly(d,l-lactic acid) (PEG-PLA) copolymer were synthesized through a self-assembly process to deliver doxorubicin and were combined to VEGF antibodies to evaluate *in vitro* and *in vivo* antitumor activity on A549 cells. When the cells were treated with 3 ug/ml of doxorubicin as free doxorubicin (control), PEG-PLA-doxorubicin, and VEGF-PEG-PLA-doxorubicin, the inhibitory effect on cell viability of VEGF-PEG-PLA-doxorubicin was the highest (10% viability), followed by PEG-PLA-doxorubicin (around 30%), and then free doxorubicin with nearly 40% viability. A nude mouse model of lung cancer induced by A549 cells was used to evaluate *in vivo* antitumor efficacy of the same particles. The tumor size of VEGF-PEG-PLA-doxorubicin and PEG-PLA-doxorubicin micelle treatment groups was significantly reduced as compared to the free doxorubicin group, with 2-fold and 1.5-fold decrease, respectively. Thus, these particles demonstrated both *in vitro* and *in vivo* improved antitumor effect, that could be potentially used for human lung cancer therapy.^106^

In a study by Ultav et al., silica biocompatible NPs (SNPs) of around 30 nm in diameter were developed with a surface modification by amination to achieve a positive charge, PEGylation conjugating to prevent opsonization, with folic acid (SNP-FA) for active targeting, further complexed with VEGF-siRNA as a gene delivery system to exploit the high amount of folate receptors on breast cancer cells. Flow cytometry and confocal microscopy revealed that folic acid conjugation enhanced the cellular uptake of MDA-MB-231 and HeLa cells. Further results obtained from ELISA tests illustrated that the VEGF gene silencing efficiency of SNP-FA displayed 73% silencing efficiency in HeLa cells and 50% efficiency in MDA-MB-231 cell lines. *In vitro* biocompatibility studies on the L929 cell line demonstrated no cytotoxicity when SNPs and nanoplexes were applied to the cells at concentrations ranging from 0.005 and 0.2 mg/mL and at ratios of 1:5–1:50 (w:w) respectively. Thus, these particles with appropriate surface modification may be suitable for gene delivery for breast cancer treatment due to affordable price, high reproducibility and good biocompatibility.^107^

siRNA-loaded chitosan (CS) NPs have been developed to suppress the proliferation and survival of cancer cells by targeting VEGF, STAT3, and Survivin. To promote the stability of these particles, cross-linkers such as dextran sulfate and poly-d-glutamic acid have been used. However, these investigations are constrained to *in vitro* and *in vivo* experiments, indicating that there is still significant progress required to apply these findings in clinical practice.^108^ Thiolated-glycol chitosan (tGC) nanocomplexes encapsulating polymerized VEGF siRNA (poly siRNAs) were developed to potentially enhance the therapeutic efficacy and overcome the resistance of bevacizumab in cancer therapy. The nanocomplexes contributed to increased structural flexibility and negative charges. The thiol-modification of glycol chitosan facilitated the stabilization of the nanocomplexes through disulfide cross-linking, alongside charge interactions. *In vivo* biodistribution studies revealed that poly siRNA/tGC NPs primarily accumulated in the tumor tissues and thus suppressed VEGF gene expression and tumor growth without leading to severe side effects. The effective targeting of tumor by poly siRNA/tGC NPs may be credited to the small size, flexibility, and deformability of the particle structure. Poly siRNA/tGC treated tumors exhibited 34.4% reduction in VEGF expression. However, when the combination treatment of VEGF siRNA and bevacizumab was provided, 43.5% of reduction in VEGF of gene expression was obtained.^109^

**Figure 5:**
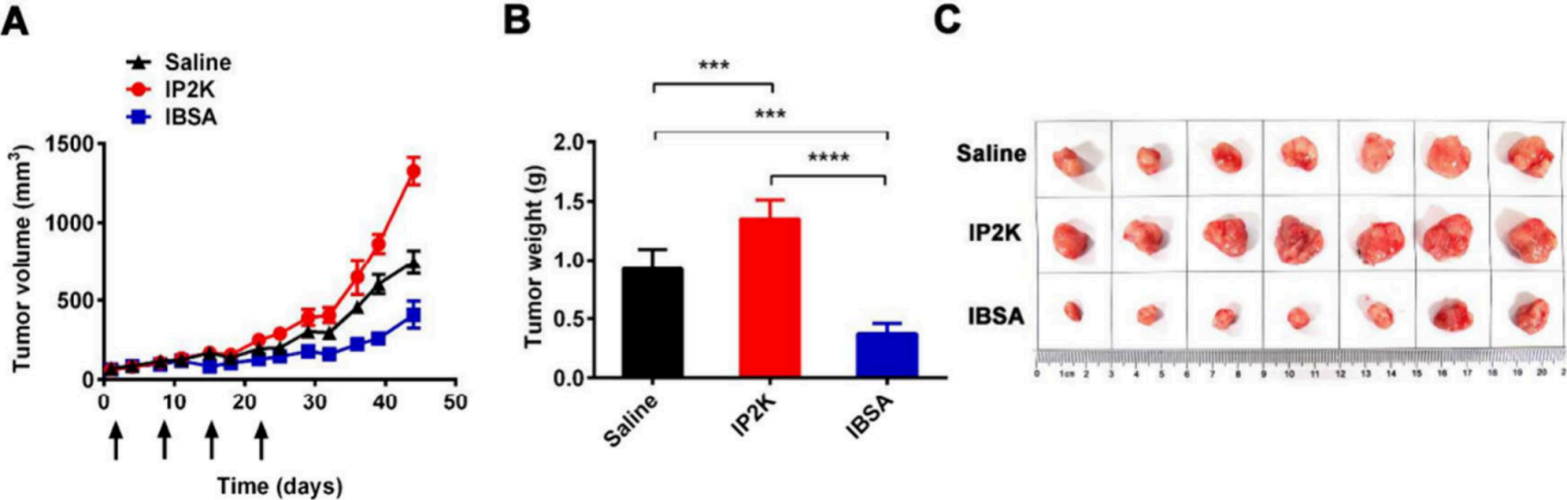
Comparison of IP2K and IBSA nanoparticle formulations in a patient-derived xenograft NSCLC model. Over 44 days, IBSA significantly inhibited tumor growth, whereas IP2K accelerated it. (A) Tumor growth curves (mean ± SEM, *n* = 7). Efficacy is shown relative to formulations due to altered pharmacokinetics. Arrows indicate injections of saline, IP2K, and IBSA. (B) Tumor weight at day 44. ***P < 0.005, ****P < 0.0001 (paired Student’s *t* test). (C) Macroscopic images of tumor tissues. Adapted with permission from ref (110). Copyright 2017 American Chemical Society.

In another study by Zhang et al., two NP formulations of itraconazole (ITA) were synthesized for intravenous use, one utilizing PEG-PLA micelles (termed IP2K) and the other involving albumin complexation (referred to as IBSA). The comparisons were performed between these two formulations *in vitro* for antiproliferation action against A549 NSCLC cells and *in vivo* by examining systemic pharmacokinetics in rats and tumor inhibition in a patient-derived NSCLC xenograft (PDX) model. When treated with increasing doses from 0.25 to 32 μM, the proliferation of A549 cells was inhibited only at ITA concentrations of 2 μM and higher. At lower concentrations, ITA had no effect, indicating that both IP2K and IBSA have limited direct effects on cancer cells themselves. The PDX mice received weekly intravenous injections of IP2K or IBSA NPs at a dose of 15 mg/kg of ITA, administered four times. Over 44 days, the PDX tumor growth exhibited contrasting trends compared to the saline-treated control group. IBSA effectively inhibited tumor growth, whereas IP2K significantly accelerated it (Figure 5). IBSA also demonstrated a more favorable therapeutic window for potential antiangiogenesis therapy due to its optimal pharmacokinetic properties, with no or minimal hematologic toxicity observed. Given its excellent safety profile, IBSA is a promising new angiogenesis therapy, offering an alternative to existing medications like bevacizumab.^110^

In a study, Li et al. investigated the therapeutic potential of systemic delivery of lentivirus-transfected mesenchymal stem cells (MSC) that are engineered to secrete soluble fms-like tyrosine kinase-1 (sFlt-1) for antiangiogenesis treatment. Mesenchymal stem cells were used as nanocarriers for the sFlt-1 gene, targeting vascular endothelial growth factor (VEGF)-driven angiogenesis in hepatocellular carcinoma. The study revealed that delivery of sFlt-1-secreting MSCs significantly inhibited tumor growth and angiogenesis while preserving normal vascular functions in nontumoral tissues. Importantly, the treatment selectively suppressed VEGF levels within the tumor microenvironment, rather than migrating to other organs like the heart and spleen, minimizing the systemic side effects. These findings highlight the potential of MSC-mediated sFlt-1 gene therapy as a targeted and safe strategy to inhibit tumor angiogenesis and promote effective cancer therapy.^111^

### 4.2 Tie2/Angiopoietin-1

Tie2 is a receptor tyrosine kinase, primarily expressed on ECs, and is a pro-angiogenic factor. It has been lately identified as a pivotal target of vascular normalization in cancer.^5^

In a novel study, ferritin-based protein cage protein C NPs (PCNs) were synthesized and tested using Lewis lung carcinoma allografts and mouse mammary tumor virus-polyoma middle tumor-antigen (MMTV-PyMT) spontaneous breast cancer models to study the antitumor and antimetastatic effect of these PCNs, and evaluate the PCN-mediated structural and functional vascular difference in the core of the tumor. It was revealed that these PCNs could significantly reduce the hypoxic area and increase the blood perfusion of tumor vessels by targeting Tie2 and EC protein C receptor/protease-activated receptor-1 (PAR-1) concurrently. Moreover, it was also found that the PCNs demonstrated excellent delivery efficacy to tumor mass and different organs. The PCNs also did not induce any hemorrhage in the internal organs or tumor masses. qRT-PCR studies revealed that Tie2 siRNA had the capability to suppress gene expression with 90% efficacy at 48-h post transfection. Thus, the study concluded that PCNs improved the normalization of abnormal tumor vessels and reduced the hypoxic areas in the tumor thus resulting in the suppression of tumor growth.^112^

A dual-responsive amphiphilic peptide mPEG1000-K (DEAP)-AAN-NLLMAAS that spontaneously assembled into NPs (P-T4) was designed with the ability to align the antitumor hydrophobic peptide T4 internally. This nanosystem was selected to improve the solubility, bioavailability, circulation time, and enzymatic degradation of peptide T4. Upon being exposed to the acidic TME, the P-T4 NPs swelled and subsequently uncovered the legumain cleavage site, finally releasing the peptide T4 that interacts with Tie2 on the surface of Tie2-positive macrophages (TPMs) and ECs. The locally released T4 affected the signaling in TPMs and ECs, preventing the relapse of tumor after chemotherapy by obstructing restoration of the blood vessels. The peptide was incorporated into the NPs to shield it from being taken away during the circulation, thus enhancing the half-life and bioavailability of the drug. The study concluded that this therapy could be employed to prevent recurrence of breast cancer postchemotherapy.^113^

In another study by Hattori et al., cationic liposome/siRNA complexes (lipoplexes) obtained using variations of cationic cholesterol derivatives or dialkyl or trialkyl cationic lipids with dioleoyl phosphatidylethanolamine (DOPE) and Tie2 siRNA load were tested for siRNA delivery and biodistribution *in vivo*. 50–80% knockdown of gene expression was observed when assayed in terms of luciferase activity. Some of the cationic liposomes were found to accumulate in the lungs while others accumulated in the liver, and some in both, thus indicating that the biodistribution is affected by the type of cationic lipid present in the liposomes.^114^

### 4.3 HIF-1α and HIF-2α

Hypoxia-inducible factors (HIFs) are dimeric proteins that play an important role in the body’s response to hypoxia or low oxygen conditions, which are characteristic of tumor tissues. Hypoxia conditions also induce a chemotherapeutic resistance phenotype. Induction of this factor leads to the overexpression of CD73 due to its specific HIF binding region, resulting in heightened cell proliferation and vascularization in tumor tissues. Thus, targeting the expression of both these proteins could provide a novel therapeutic approach.^115^

Superparamagnetic iron oxide (SPION) NPs encapsulated in thiolated chitosan and trimethyl chitosan and loaded with the siRNA against both HIF and CD73, were developed as a potential nanocarrier with the TAT peptide conjugated to enhance the cellular uptake in cancer cells. When the cytotoxicity of the above NPs was tested on B16-F10, 4T1, and CT26 cell lines, those with HIF-1α-siRNA were found to be more cytotoxic as compared to the CD73siRNA-carrying NPs, while the set with both HIF-1α and CD73-siRNA load showed an enhanced cytotoxic effect compared to single treatments. Real-time quantitative polymerase-chain reaction (RT qPCR) analysis showed significant inhibition of the targeted protein expression, with the combination of the two siRNAs showing the highest effect. Hypoxia-induced angiogenesis was evaluated using the chorioallantoic membrane assay and, interestingly, tumor growth and blood vessel formation were inhibited by the NPs along with the suppression of other pro-angiogenic factors such as TGF-β, VEGF, and FGF.^116^

**Figure 6:**
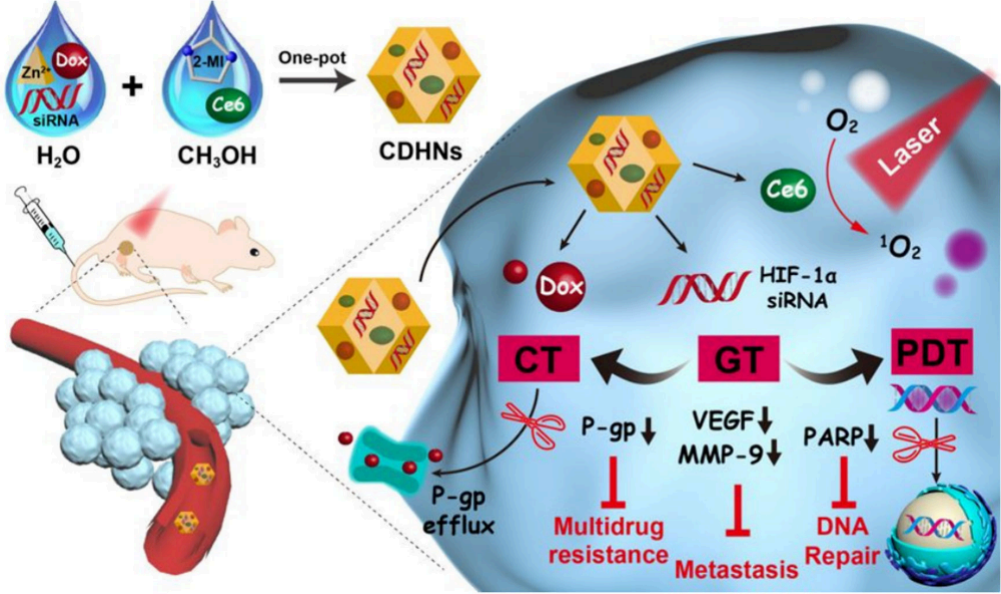
siRNA-based therapy against HIF-1α using biodegradable metal–organic frameworks for overcoming chemophotodynamic therapy resistance in hypoxic tumors. Reproduced with permission from ref (117). Copyright 2020 American Chemical Society.

siRNA-based therapy against HIF-1α has also been applied to overcome the resistance to chemophotodynamic therapy, evident in hypoxic tumor environments. Biodegradable nanoscaled metal–organic frameworks (NMOFs) have been explored for encapsulation of siRNA due to their high porosity, large specific surface area, and pH–responsiveness. A one-pot synthesis method was followed to generate the zinc-based, pH-responsive ZIF-8 NPs of around 180 nm diameter containing Ce6 (for photo irradiation), doxorubicin, and HIF-1α-siRNA, and tested in breast cancer models *in vitro* and *in vivo* (Figure 6). The NPs were found to be biocompatible with stabilization and uptake of the photosensitizer Ce6 into the lysosomes, as compared to free Ce6; further photo irradiation promoted the lysosomal escape of the therapeutic. The siRNA internalization and prevention of nuclease degradation were facilitated by the NPs with 50% inhibition of HIF-1α expression obtained, thus proving it as a promising anticancer platform.^117^ Similar azoreductase-responsive functional MOFs were designed with chemotherapeutic and siRNA load against HIF-1α. The azoreductase framework gave specific release in the hypoxic environment, along with decreasing the efflux of the chemotherapeutic drug, resulting in inhibition of chemoresistance. Inhibition of expression of HIF-1α, MDR1, and p-glycoprotein was observed in MCF-7 cell lines and mice bearing MCF-7 tumors. Fluorescence imaging of kidneys, spleen, heart, liver, spleen, and lung revealed greater accumulation of the NPs in the tumor regions compared to other organs.^118^

Micro-RNA is another route applied to obtain suppression of pro-angiogenic factors. In one study by Zhang et al., miR-320a (which is reported to directly bind and regulate the mRNA of HIF-1α) encapsulated inside extracellular vesicles was released from cancer-associated fibroblasts (CAFs) to prevent endometrial cancer progression. Fibroblasts are known to be involved in cancer processes and EVs derived from CAFs can alter tumor phenotype.^119^ EVs secreted from CAFs overexpressing miR-320a were found to suppress the viability of endometrial cancer cells as well as downregulate HIF-1α expression.^120^ The micro-RNA miR-519c is downregulated, while HIF-1α is overexpressed aberrantly in pancreatic cancer, which further results in hypoxia-induced chemoresistance. Transfection of miR-519c along with 14% gemcitabine payload into a redox-sensitive polymer mPEG-*co*-P(Asp)-*g*-DC-*g*-S-S-GEM, inhibited HIF-1α and ABCG2 (with 90% release of gemcitabine in the presence of L-glutathione), resensitized resistant cancer cells to gemcitabine, thus resulting in decreased desmoplasia. Cytotoxicity, apoptosis, and efficacy were tested in MIA PaCa-2R spheroid culture to emulate the hypoxic environment and significant downregulation of HIF-1α was obtained in Western Blot analysis.^121^

Downregulation of HIF-1α using the CRISPR/Cas9-based editing system is a recently explored, novel system. A nanocarrier was designed using R8-dGR peptide modified cationic liposomes, harboring the plasmids encoding Cas9 and HIF-1α-targeting sgRNA as well as PTX load. The presence of the R8-dGR peptide enhanced the cellular uptake of liposomes on BxPC-3 pancreatic cancer cells. In addition to HIF-1α, downregulation of the downstream VEGF and MMP-9 was observed, promoting an antimetastatic effect. Blocking of HIF-1α also had a positive effect on the antitumor and antiproliferative activity exhibited by PTX in a BxPC-3 xenograft model.^122^

Resistance to radiotherapy is a major problem in cancer treatment due to the creation of hypoxic areas in malignant tumors, which render radiotherapy ineffective. Dual-mode endogenous (through HIF-1α silencing) and exogenous (through the gold component) nanosensitizers based on dendrimer-entrapped gold NPs have been developed with a HIF-1α siRNA load. G5 PAMAM dendrimers partially modified with 1,3-propanesultone creating zwitterionic moieties were used as a template for Au-NP synthesis using sodium borohydride reduction chemistry. When tested *in vitro* in A549 lung cancer cells and *in vivo* in mice bearing A549 tumors, these NP/siRNA polyplexes were capable of successful knockdown of HIF-1α and its downstream genes. Generation of ROS was found to be enhanced along with double strand DNA damage resulting in the alleviation of radioresistance.^123^

### 4.4 Transforming Growth Factor-β (TGF-β)

TGF-β is a secreted cytokine that is associated with dynamic crucial processes, namely homeostasis, tissue repair, inflammation, immune responses, cell growth, differentiation, proliferation, apoptosis, and tumor angiogenesis. The TGF-β signaling pathways are deregulated in tumor cells, and as a result it becomes unable to control proliferation.^124^

In breast cancer, TGF-β signaling gets hyper activated thus promoting cancer progression and metastasis. In work by Zhang et al., a particular type of bioresponsive NPs-rotaxane-doxorubicin-heparin-SB431542 (R (D)/H(S) NPs), were designed by β-cyclodextrin-grafted heparin and pH-responsive pseudorotaxane to produce enhanced chemotherapeutic efficacy on breast cancer via TME modulation. It was found that when doxorubicin and TGF-β receptor inhibitor (SB431542) were loaded in the R (D)/H(S) NPs, a rapid release was obtained as a result of low pH in endosomes and heparanase (HPSE) in TME. Tumor-associated fibroblasts (TAFs) are the key components of TME and serve as a source of TGF-β and collagens. Thus, blocking of TAFs production could be a well-regulated method in remodelling the TME to improve chemotherapy. It was found that R (D)/H(S) NPs blocked the formation of TAFs and decreased the secretion of TGF-β. The anticancer efficacy of R(D)/H(S) NPs was determined *in vitro* using 4T1 cells and *in vivo* in female BALB/c mice. It was found that these NPs provided tumor inhibition, reducing TAFs, immune activation, regulation of TME, and eventually improving the chemotherapeutic efficacy of doxorubicin. Summarizing, these particles could be employed to improve the chemotherapeutic efficiency and serve as a novel therapy for future breast cancer treatments.^125^

In another study, PEI-modified carboxyl-styrene/acrylamide (PS) copolymer nanospheres were designed as a method of delivery of unmethylated cytosine-phosphate-guanine (CpG) oligodeoxynucleotides and TGF-β receptor inhibitors for cancer treatment. The TGF-β receptor blockers, namely, LY2157299, were captured in the PS through hydrophobic interactions, whereas CpG oligodeoxynucleotides were encapsulated in the PS via electrostatic interactions. *In vivo* studies concluded that PS-LY/CpG treatment inhibited the tumor by up to 99.7% without causing any significant toxicity compared to the control group. This could have been due to the activation and amplification of the T-cells in the BALB/c mice (male, 4–6 weeks) model. When CpG and TGF-β are codelivered, the additive effect results in enhanced cancer therapeutic efficacy. Thus, PS-LY/CpG may be a good candidate for liver cancer and other tumor therapies.^126^ The NPs were functionalized with CGKRK peptide to achieve targeted biodistribution in tumor sites through overexpression via heparan sulfate proteoglycan binding in the TME. *In vivo* imaging system (IVIS) spectrum optical imaging device was used to study the tumor-targeting capacity of NP-CGKRK in orthotropic pancreatic tumor sites. It was found that these particles exhibited stronger fluorescence intensity at tumor tissue in less than 24 h after intravenous injection in tumor-bearing nude mice as compared to the NPs without the peptide. In particular, the concentration of NP-CGKRK at the tumor locations was approximately 3.3 times higher than that of NP after 24 h. This demonstrated that the CGKRK peptide had a higher ability to improve the distribution of PEG-PLA NPs in the orthotropic pancreatic tumor tissues. The toxicity study revealed that both Frax-NP-CGKRK and siKRAS-LCP-ApoE3 produce an ideal safety profile making this combination a favorable candidate for the treatment of pancreatic cancer.^127^

### 4.5 Other Pro-angiogenic Factors

Several other factors that influence angiogenesis have been modulated by combining gene-based therapy with NP systems for effective strategies to combat cancer using nonviral vectors. An interesting class are microRNAs (miRNAs) such as miR-141, which has tumor suppressive and antiangiogenic effects, or miR-181a, which is overexpressed in cancer.^128^ Chitosan/miR-141 nanoplexes were prepared using a complexation method, and different weight ratios of chitosan were tested with a fixed amount of miRNA. When these nanoplexes were delivered into MDA-MB-231 and MDA-MB-435 cells (which displayed lower endogenous levels of miR-141), miR-141 levels were found to reach that of MCF-10A, which is a nontumorigenic cell line. Administration of the NPs had a diminishing effect on the levels of pro-angiogenic VEGF, epithelial–mesenchymal transition mediated by E-cadherin, and metastatic colonization (determined by Igfbp-4 and Tinagl1 levels) and invasion, while having a remarkable increase in apoptosis. This study thus proved the tumor suppressive role of miR-141 and the developed chitosan/miR-141 nanoplexes hold an innovative strategy for breast cancer treatment.^129^

On the other hand, miR-181a is an oncogenic miRNA that promotes tumor progression. The target of miR-181a is the Regulator of G-protein signaling 16 (RGS16), whose inhibition results in an increase of the levels of MMP1 and VEGF, affecting metastasis and angiogenesis. In a novel delivery approach, anti-miR-181a oligonucleotides were combined through charge interactions with Janus base nanotubes (consisting of the biomimetic molecule 6-amino fused adenine and thymine), forming an NP delivery platform. When transfected into human chondrosarcoma cell lines CS-1 and mice xenograft models, both MMP1 and VEGF expression levels were found to decrease, with an increase in cell survival and decrease in metastatic rates. However, angiogenesis was not significantly affected.^130^

## 5 Nanotechnology-Based Combination Therapies

Despite many successful preclinical outcomes (Table 1), clinical phase I/II trials demonstrate that the majority of antiangiogenic monotherapies were unable to significantly reduce tumor growth. A possible explanation for this inefficiency may be a) the difference in drug sensitivities between cancer growth pattern in humans and mice; the tumor develops quicker in mice as compared to humans; b) angiogenesis may not be linked to the growth or regrowth of human tumors; the proliferation of tumor cells may be adequately supplied via vascular mimicry and pre-existing host vessels; c) angiogenic pathways are distinct; bFGF or other factors are most likely involved in humans, whereas VEGF is predominant in mice; or d) therapeutic resistance may arise from hypoxia-inducible factors and the genes that respond to their effects in angiogenic therapy. These challenges may be addressed by combining the antiangiogenic drug with conventional chemotherapy, radiation therapy, phototherapy, and immunotherapy; it is adequate to anticipate that several treatments will be successful in tandem. Antiangiogenic therapy should increase drug delivery by lowering interstitial fluid pressure at the tumor location, which aids combination therapy. Radiation therapy (RT) and photodynamic therapy (PDT), which depend on the production of ROS disrupting regular biomolecule functions, may be greatly improved by increased intratumoral tissue oxygenation achieved by vascular normalization.^131^ Lately, a significant number of studies have been published in this area, and the following subsection covers some different combination therapies.

**Table 1:** Nanotherapeutic System Applications in Various Preclinical Disease Models^a^.

Factor	Nanosystem	Drug	Disease	Model	Highlights	Ref.
Antiangiogenic factors						
Endostatin	Tandem peptide TAT (Transactivating Transcriptional Activator)-AT7-modified PEI (polyethyleneimine) with heterobifunctional PEG (polyethylene glycol) linker (nanosystem referred as PPTA)	Angiogenesis-inhibiting secretory endostatin gene (pVAXI-En)	Glioblastoma	Orthotopic U87-mCherry-luc glioma-bearing nude mice	•Increased binding efficiency (VEGFR-2 and NRP-1) and cellular uptake of TAT-AT7 in ECs (5-fold and 119-fold increase) compared to TAT and AT7 alone	(65)
					•PPTA/pVAXI-En reported stronger BBB and BTB penetrating abilities, increased selective glioma targetability	
					•PPTA/pVAXI-En inhibited glioma growth as well as significantly reduced microvessel density	
	Polymeric PEI-PEG-chitosan-grafted nanosystem	LyP-1 (a homing peptide)	Squamous cell carcinoma (P32 receptor)	Athymic nude mice with a KYSE-30 cell xenograft	•LyP-1-modified nanosystem showcased decreased cell proliferation and migration, as well as restricted VEGF-C and MMP2 expression	(66)
					•2-fold decrease in tumor volume reported with endostatin-loaded NPs in comparison with native endostatin	
	Polymer (poly (ethyl acrylate-co-butyl methacrylate-co-methacrylic acid) polymer) -quantum dots micelle (QDs-M-MS) loaded with rhES (system referred as rhES-QDs-M microspheres (rhES-QDs-M-MS))	Recombinant human endostatin (rhES)	Cancer (lung-targeted)	Lung cancer bearing mice model	•rhES-QDs-M-MS exhibited good, sustained release behavior (at least 15 days)	(67)
					•Improved antitumor activity demonstrated by rhES-QDs-M-MS than control and rhES group with significant decrease in tumor volume and weight	
	Hyaluronic acid-tyramine hydrogel (HA-Tyr)	Endostatin (ES)	Lung Cancer	Lewis lung cancer (LLC)-bearing C57 female mice model	•ES/HA-Tyr significantly decreased tumor volume and weight compared with that in ES (*p* < 0.01), while ES/HA-Tyr + Radiation (*p* < 0.01) led to a stronger tumor growth inhibition compared to either monotherapy	(70)
					•Improved serum concentration of ES was observed from ES/HA-Tyr in tumor sites compared to ES alone ((*p* < 0.01). ES/HA-Tyr also significantly decreased the peripheral concentration indicating increased local drug administration in tumor	
					•ES/HA-Tyr + Radiotherapy significantly increased pericyte coverage (*p* < 0.05) in comparison to other therapies thus promoting vascular normalization inhibiting angiogenesis	
IL-12	CuS NPs within SiO_2_ pores and PDMAEMA polycation on the SiO_2_ surface (nanosystem referred as CSP)	IL-12 gene (CSP@IL-12)	Melanoma	Murine melanoma B16F10 tumor bearing C57BL/6 mouse model (male)	•Photothermal agent and gene codelivery was inhibited primary local tumor as well as residual and abscopal tumor. improved antitumor effects	(87)
					•Promotes proliferation and infiltration of CD8+ T cells improving the systemic immune response	
	PEG-PEI-cholesterol lipopolymer delivery system (nanosystem referred as PPC)	GEN-1 (*IL-12* gene expression plasmid with a synthetic plasmid delivery system)	Ovarian cancer	SKOV3 human ovarian cancer bearing ID8 C57BL/6 mouse model	•5-fold reduction in VEGF levels demonstrated after first day of treatment injection with response lasting for at least 3 days	(92)
					•Combination therapy of PPC with carboplatin and paclitaxel reported prolonged survival	
	DOTAP/MPEG_2000_–PDLLA_4000_–MPEG_2000_ modified by cationic liposome 1,2-dioleoyl-3-trimethylammonium propane (DOTAP) (nanosystem referred to as DOTAP/MPEG_2000_–PDLLA_4000_–MPEG_2000_ (DMPM))	Interleukin (IL)-12 plasmid (pIL-12)	Colon cancer and melanoma	CT26 tumor bearing BALB/c female mice (colon cancer) and B16-F10 tumor bearing C57BL/6 female mice (melanoma)	•pIL-12/DMPM inhibited the tumor growth by inhibiting the tumor cell proliferation as well as significantly decreased the tumor nodules in treated group	(97)
					•pIL-12/DMPM treatment inhibited tumor angiogenesis and induced tumor cell apoptosis	
Pro-angiogenic factors						
VEGF	Polymeric NPs	Paclitaxeland VEGF-siRNA (small interfering ribonucleic acid)	Breast cancer	4T1^LUC^ breast cancer mouse orthotropic model	•PTX-siRNA-VEGF-NPs showcased most tumor suppressive effects and significantly inhibited angiogenesis, better than the PTX-NPs and siRNA VEGF-NPs groups	(106)
	Biodegradable PEG-PLA micelles (Nanosystem referred to as VEGF-PEG-PLA-DOX micelles)	DOX and VEGF antibodies	Lung cancer	A549 Lung cancer bearing nude mouse model	•VEGF-PEG-PLA-DOX micelles significantly reduced the tumor cell mass and volume than the PEG-PLA-DOX micelles and free DOX	(108)
					•VEGF-PEG-PLA-DOX micelles induced faster ROS release triggering cell apoptosis	
	Thiolated-glycol chitosan (tGC) nanocomplexes (Nanosystem referred as psi(VEGF)/tGC)	Polymerized VEGF siRNA (poly-VEGF siRNAs	Epidermoid carcinoma	A431 tumor bearing BALB/c male nude mice model	•psi(VEGF)/tGC NPs demonstrated suppression of 34.4% in VEGF expression	(111)
					•A 43.5% remarkable reduction of VEGF gene expression with combination treatment of VEGF siRNA and bevacizumab	
	Polymer micelles (IP2K) and albumin NPs (IBSA)	Itraconazole	Nonsmall cell lung cancer	NSCLC patient-derived xenograft mice model (NOD/SCID/IL2λ-receptor null mice and BALB/c nu/nu mice)	•IBSA retarded tumor growth while IP2K accelerated the tumor growth which was attributed to the formulation dependent PK	(112)
					•IP2K reported elevated *C*_max_ values which induced tumor hypoxia through a strong angiogenesis inhibition leading to an aggressive tumor growth	
	Lentivirus-transfected mesenchymal stem cells (MSC) secreting sFlt1 (Nanosystem referred to as LV-sFlt-1-MSCs)	Soluble form of the VEGF receptor 1 (sFlt1)	Hepatocellular carcinoma	SMMC-7721 tumor-bearing male athymic BALB/c nude mice model	•LV-sFlt-1-MSCs reported inhibition of *in vitro* tube formation, tumor growth, and prolonged survival time of tumor-bearing mice	(113)
					•LV-sFlt-1-MSCs showcased a significant decrease in microvessel density in the treatment group vs control group (38.3 ± 1.7 vs 96.1 ± 2.0) demonstrating inhibition of angiogenesis	
					•It also inhibits cell proliferation, as the reported average proliferation index of 53.7 ± 2.3% in the group treated with LV-sFlt-1-MSCs in comparison to the 86.9 ± 1.3% in the control group	
Tie2/Ang1	Cationic liposome some/siRNA complexes (lipoplexes)	Protein kinase N3 (PKN3) siRNACont. siRNA	Lung cancer	Lewis lung carcinoma bearing female C57BL/6N mice model	•The type of cationic lipid utilized in the liposome preparation influencing the biodistribution of siRNA	(116)
					•DC-1-16/DOPE or DC-1-18/DOPE liposomes show promising results for siRNA drug delivery to lungs and further suppression of targeted gene	
HIF-1α and HIF-2α	pH-responsive zeolitic imidazolate framework-8 (ZIF-8) NPs (CDHNs)	chlorin e6 (Ce6), doxorubicin (DOX), and HIF-1α siRNA	Breast cancer	MDR/MCF-7 tumor-bearing BALB/c female nude mice model	•CDHNs demonstrated the tumor-specific targetability and pH-responsive behavior at the tumor site	(119)
					•CDHNs combined with the gene-photochemotherapy silenced the HIF-1α gene resulting in improved antitumor and antimetastatic capability	
	Generation 5 poly(amidoamine) dendrimers modified with 1,3-propanesulton with Au NPs (Nanosystem referred as Au DENPs)	HIF-1α siRNA	Lung cancer	A549 tumor bearing mice model	•The combined exogenous (Au) and endogenous (HIF-1 α siRNA) components significantly improved the radiotherapy showcased by the improved ROS generation, cell apoptosis (60% more than control group) and tumor necrosis	(125)
TGF-β	Rotaxane-doxorubicin-heparin-SB431542 (R (D)/H(S) NPs)	Doxorubicin and TGF-β receptor inhibitor (SB431542)	Breast cancer	Mix of 4T1 and 3T3 cell tumor bearing female BALB/c mice model	•R (D)/H(S) NPs reported inhibition of TAFs production and decreased TGF-β secretion	(127)
					•It significantly inhibited tumor growth (65%) and demonstrated antimetastatic behavior marked by raised CD8^+^ and CD4^+^ T cell levels	
	PEI-modified carboxyl-styrene/acrylamide (PS) copolymer nanospheres	Unmethylated cytosine-phosphate-guanine (CpG) oligodeoxynucleotides and TGF-β receptor inhibitor (LY2157299)	Hepatocarcinoma	H22 tumor bearing male BALB/c mice model	•Co delivery of LY and CpG via PS nanosphere treatment revealed a striking 99.7% tumor inhibition rate compared to PS-LY alone group. This synergistic effect could be attributed to the CD8+ T-cell immune responses	(128)
	CGKRK-modified NPs (Frax-NP-CGKRK) and lipid-coated calcium phosphate (LCP) biomimetic NPs (siKras-LCP-ApoE3)	Antifibrotic fraxinellone and siRNA drugs (siKras)	Pancreatic cancer	Orthotopic pancreatic tumor bearing nude mice model	•Frax-NP-CGKRK reversed the CAFs to the dormant state leading to reduced immunosuppression and increased blood perfusion	(129)
					•This allowed the distribution of siKras-LCP-ApoE3 into the tumor vasculature resulting in KRAS gene silencing and thus damage to tumor cells	

a The table summarizes various nanotherapeutic systems applied in different preclinical models targeting specific cancer type and highlights the significant research findings.

By combining an antiangiogenic agent (TNP-470), chemotherapeutic drug DOX, near-infrared II fluorescent Ag2S quantum dots (QDs), recognition peptide cyclic RGD (cRGD), and PEG, Song et al. developed a vascular-targeted delivery system known as T&D@RGD-Ag2S. *In vitro*, the T&D@RGD-Ag2S NPs were more effective than the free drug at preventing the ECs to form vessels. These NPs accumulated quickly at tumor sites due to its high specific binding ability and prolonged blood circulation time. This NP system effectively suppressed tumor growth *in vivo* because of combined antiangiogenetic effects from TNP-470 and widespread tumor apoptosis produced by DOX.^132^

The hypoxic TME reduces tumor cell sensitivity to radiation, resulting in inefficient radiotherapy. To address this issue, antiangiogenic drugs promote the radiation response by mechanisms such as increased tumor oxygenation, decreased vascular density, and radio-sensitized ECs. Several studies found that radiation therapy can restore the vasculature. Clément-Colmou et al. compared various radiation fractionation regimens and finally observed that high-dose fractions reduced hypoxia and improved vascular functionality in the tumor microenvironment.^133^ Lan et al. also discovered that hypo-fractionated radiation (HFRT) could reduce vascular density while normalizing tumor vasculature.^134^ It is unclear whether properly fractionated radiation combined with other antiangiogenic therapy is more successful than radiation alone, and how vascular-optimized radiation should be used in combination with other treatments.

Due to noninvasiveness, less chances of resistance development, easily tuneable intensity, exposure time, dose intervals, and high selectivity, phototherapy including photodynamic therapy (PDT) and photothermal therapy (PTT), has gained great attention.^135^ The collective action of light, oxygen, and photosensitizers in PDT can produce ROS, resulting in irreversible cytotoxic cell death. Although PDT has been successful in the treatment of superficial bladder cancer, obstructive lung cancer, and skin cancer over the last few decades, some critical barriers, including hypoxia, short half-life (<40 ns), and small diffusion radius (220 nm) of ROS, seriously hinder PDT efficacy.^136^ In PTT, photothermal agents cause cellular hyperthermia through light absorption, resulting in thermal ablation of cancer cells. Mild temperatures between 41 and 48 °C can induce apoptosis, while temperatures exceeding 48 °C can induce irreversible apoptosis. To date, a wide range of PTT materials have been investigated.^137^ According to Yang et al., effective cancer treatment can be achieved by using a functional drug delivery system consisting of liposomes that contain an antiangiogenic drug and the NIR dye IR780. As the IR780 concentration increased *in vitro*, the survival of light-radiated cells declined but laser-induced apoptosis increased. Following laser irradiation, Lip-IR780-sunitinib presented an inhibitory effect equal to or higher than free sunitinib. In a syngeneic antitumor treatment conducted using 4T1 tumors in mice, the treatment was more effective at inhibiting tumor growth *in vivo* than controls based on IR780 or sunitinib in free or liposomal form. Finally, a CD31 staining experiment revealed that the vessel density in the Lip-IR780-Sunitinib/laser group was lowest.^138^

The vascular endothelium plays a major role in the immunological response to malignancies. Abnormal tumor vessels and protumoral immune cells form a vicious circle that substantially impairs anticancer immunity and promotes tumor proliferation: abnormal tumor vessels enhance protumoral immune cell invasion that promotes tumor-associated angiogenesis. As a result, halting this process with antiangiogenic therapy could be a promising strategy for enhancing anticancer immunity and overcoming drug resistance.^139,140^ Magnussen et al. reported that antiangiogenic drugs could temporarily restore tumor vasculature, hence enhancing cancer therapy and drug delivery to tumor tissues. Vascular normalization resulted in a number of additional effects, including an enhanced oxygen supply, alterations in infiltrating cells, and a reduction in abnormal vessel porosity. However, these effects are dose-dependent; using excessive or insufficient antiangiogenic drug may be detrimental.^141^ Kikuchi et al. demonstrated the improved therapeutic outcome of integrated immunotherapy and antiangiogenic therapy, highlighting their potential benefits.^142^ Antiangiogenic therapy coupled with immunotherapy targeting the T-cell checkpoint receptor PD-1 has shown promise in the treatment of NSCLC. Thus, antiangiogenic drugs may stimulate the immune response, while immunotherapies can potentially also have antiangiogenic effects.^143^

## 6 Clinical Trials

Clinical trials are exploring nanotherapeutic and combination approaches for various cancers (Table 2). A one-arm study at Henan Cancer Hospital (2019–2020) evaluated the combination of apatinib with albumin-bound paclitaxel for platinum-resistant recurrent ovarian cancer.^144^ Another phase Ib/II trial currently recruiting has investigated nanotherapeutics, antiangiogenics, and immunotherapy for unresectable pancreatic cancer, combining nab-paclitaxel, gemcitabine, surufatinib, and KN046 (a bispecific PD-L1/CTLA-4 antibody), showing promising safety and antitumor activity so far.^145^

**Table 2:** Nanotherapeutic System Applications in Clinical Trials for Combination Therapies^a^.

Cancer Type	Trial Phase	Treatment Combination	Key Findings/Aims	Trial ID (ClinicalTrials.gov)	Ref.
Ovarian Cancer (Platinum-resistant recurrent)	One-arm study (2019–2020)	Apatinib + Albumin-bound Paclitaxel	Combination improves clinical outcomes	NCT03942068	(144)
Pancreatic Cancer (Unresectable)	Phase Ib/II (Recruiting)	Nab-paclitaxel + Gemcitabine + Surufatinib + KN046 (PD-L1/CTLA-4 antibody)	Promising safety and antitumor activity	NCT05832892	(145)
Non-Small Cell Lung Cancer	Active trial	Quaratusugene Ozeplasmid (Reqorsa, nonviral lipid NP gene therapy) + Pembrolizumab	Dose escalation phase followed by randomized comparison with standard treatments	NCT05062980	(146)
Gastric Cancer	Phase I/II	Liposomal Irinotecan (MM-398) + Ramucirumab (VEGFR2 antagonist)	Showing potential efficacy	NCT03739801	(147)
Ovarian, Fallopian Tube, or Peritoneal Cancer (Platinum-resistant)	Completed	Irinotecan Liposomes + Bevacizumab	Partial responses observed in refractory cases	NCT04753216	(148)
Metastatic Colorectal Cancer	Recruiting	Liposomal Irinotecan + Oxaliplatin + 5-FU/LV + Bevacizumab or Cetuximab	Evaluating first-line treatment, analyzing circulating tumor DNA and genetic mutations	NCT06225622	(149)
Metastatic Pancreatic Cancer (Postfirst-line chemotherapy failure)	Multicenter trial (Not yet recruiting)	Nal-IRI + 5-FU/LV + Benmelstobart + Anlotinib ± SBRT	Aims to improve efficacy via multimodal approach	NCT06662006	(150)

a The table summarizes various nanotherapeutic systems applied in clinical trials that explore combinatorial approaches targeting specific cancer types and highlights key findings so far.

In nonsmall cell lung cancer, quaratusugene ozeplasmid (Reqorsa), a systemic gene therapy delivered by nonviral lipid NPs, was combined with pembrolizumab to enhance immune activation and tumor suppression. The active trial has a dose escalation phase followed by randomized comparison with standard treatments.^146^

For gastric cancer, a phase I/II trial combined liposomal irinotecan (MM-398) with ramucirumab, a VEGFR2 antagonist, showing potential efficacy.^147^ Similarly, a trial for platinum-resistant ovarian, fallopian tube, or peritoneal cancer tested irinotecan liposomes with bevacizumab to improve outcomes in refractory cases, with partial responses observed.^148^ In metastatic colorectal cancer, a recruiting dose escalation and expansion trial will evaluate liposomal irinotecan with oxaliplatin, 5-FU/LV, and either bevacizumab or cetuximab as first-line treatment, including an analysis of circulating tumor DNA and genetic mutations to guide treatment strategies.^149^ A multicenter trial, not yet recruiting, will explore the combination of nal-IRI, 5-FU/LV, benmelstobart, and anlotinib ± SBRT for advanced metastatic pancreatic cancer after first-line chemotherapy failure, aiming to improve therapeutic efficacy through a multimodal approach.^150^

While these examples and other combination therapies have been approved for clinical trials, antiangiogenic drugs are typically not formulated within nanosystems but are instead administered alongside other drugs in NPs such as liposomes. However, there is a significant opportunity to engineer dual-delivery nanosystems capable of codelivering both anticancer and antiangiogenic agents.^151−153^ This approach has the potential to enhance therapeutic efficacy while minimizing systemic toxicity, offering a more targeted and effective treatment strategy for cancer. The development of such dual-delivery systems warrants further exploration, as their integration could revolutionize cancer therapy. Collaborative efforts between industry and academia hold immense promise for advancing these innovative nanosystems with the aim to ultimately transform cancer care.

## 7 Limitations and Potential Solutions in Nanomedicine Development

A number of challenges such as stability, targeting specificity, toxicity, controlled release mechanisms, manufacturing scalability, regulatory hurdles, immunogenicity, limited tissue penetration, the complexity of biological interactions, and the high cost of development hinder the clinical application of nanomedicines, despite the fact that they represent a significant advancement in targeted drug delivery.^154^

First, achieving precise and selective targeting of the angiogenic vasculature is one of the foremost challenges in developing nanodrugs for antiangiogenesis. Tumor blood vessels are often characterized by abnormal vasculature, including leaky blood vessels and irregular ECs, which can make it difficult to target only tumor-associated vessels without affecting normal tissue. Antiangiogenesis strategies often target biomarkers like VEGF receptors, integrins, or angiopoietins, but these molecular targets can be highly heterogeneous within tumors and across patients.^97^ For example, not all tumors overexpress VEGF receptors, and other pathways such as FGFs can also drive angiogenesis in different tumor types. This heterogeneity means that NPs need to be designed to target specific tumor markers, which vary between individual tumors and their microenvironment.

Furthermore, tumor heterogeneity extends beyond the molecular markers. The physical properties of tumor vasculature, such as differences in permeability, also contribute to challenges in targeted delivery. For instance, while NPs may penetrate the leaky blood vessels of tumors through the EPR effect, there is significant variability depending on tumor size, tumor type, etc.^155^ Additionally, these NPs must not only target ECs but should ideally deliver antiangiogenic agents directly to the tumor’s blood vessel network without affecting normal ECs. Achieving this level of selectivity is difficult but necessary to avoid systemic toxicity and optimize therapeutic outcomes.

Another significant issue in the clinical application of antiangiogenic nanodrugs is ensuring adequate tumor penetration and bioavailability. While NPs are designed to improve the solubility and stability of hydrophobic drugs, their penetration into solid tumors remains problematic. Tumors often have dense ECMs, thickened basement membranes, and poorly formed vascular structures, which act as physical barriers to the diffusion of NPs into the tumor core. Furthermore, tumors exhibit a high interstitial fluid pressure, which can limit the ability of NPs to extravasate and reach their target within the tumor. This is especially problematic for solid tumors that lack proper vascular development, making it difficult for drug-carrying NPs to access the target angiogenic tissue effectively.^156^

Moreover, the distribution of NPs in the tumors is another concern. Even if NPs are able to extravasate into the tumor, their uneven distribution within the tumor tissue can lead to suboptimal therapeutic efficacy. Deep penetration into the tumor and sustained drug release at the site of angiogenesis are critical for inhibiting blood vessel formation effectively. However, this is particularly difficult to achieve in advanced solid tumors, which tend to have more complex and heterogeneous vasculature. This issue is exacerbated by the fact that antiangiogenic drugs may need to be delivered over a longer period to ensure sustained inhibition of angiogenesis, further complicating the delivery of NPs to the tumor.^157^

On the other hand, achieving the desired drug release profile is another bottleneck in the clinical translation of nanodrugs for antiangiogenesis. For antiangiogenic therapies, it is crucial that the therapeutic agents are released in a controlled, sustained, or triggered manner at the tumor site. However, the controlled release mechanisms of many NPs are still under development. Many nanocarriers are designed to respond to environmental stimuli such as pH changes, enzymatic activity, or temperature fluctuations in order to trigger drug release at the site of disease.^158^ While this approach holds promise, fine-tuning these mechanisms for optimal release profiles *in vivo* remains a significant challenge. In some cases, NPs may release their payload prematurely or fail to release it at the tumor site, both of which undermine therapeutic potential. Furthermore, achieving a predictable and reproducible release profile can be difficult due to the complex interactions among the NP, the encapsulated drug, and the surrounding biological environment. For instance, the presence of proteins, immune cells, and other biological molecules in the bloodstream can alter the release rate of the drug.^159^ Additionally, variations in TME, such as pH levels or enzyme expression, complicate the development of “one-size-fits-all” drug release systems. Overcoming these challenges requires advanced formulations and careful selection of release triggers as well as a deeper understanding of the TME.

The biocompatibility of NPs also plays a significant role in the safety of these drug delivery systems. The size, shape, and surface properties of NPs can influence their interaction with cells and tissues. Some NPs may induce cytotoxicity in healthy cells, either through direct interactions with cellular membranes or by generating ROS, which can lead to oxidative stress.^160^ This toxicity must be carefully managed as it can negate the advantages of targeting angiogenesis. In addition, NPs are often recognized by the immune system as foreign bodies, which can lead to immune responses such as the activation of the complement system or the mononuclear phagocyte system, resulting in rapid clearance and inflammation.^159^ This can lead to lower drug bioavailability and potentially immune-related side effects. Moreover, NPs that accumulate in the liver, spleen, and kidneys over time may result in organ-specific toxicity, particularly with repeated administration, which can reduce the therapeutic index. Finding a balance between sufficient drug delivery and minimal side effects is essential for the clinical viability of these therapies.

Additionally, while NP-based drug delivery systems are often optimized in the laboratory, scaling up them for large-scale production remains a formidable challenge. Manufacturing processes for nanoformulations must be reproducible, efficient, and capable of meeting regulatory standards for clinical use.^51^ In particular, batch-to-batch consistency is critical to ensure that the properties of the NPs (e.g., size, surface charge, and encapsulation efficiency) remain consistent across different production runs. However, scaling up production techniques, such as solvent evaporation, extrusion, or emulsification, to meet clinical and commercial demands can result in variability in NP characteristics, leading to issues in therapeutic efficacy and safety. The manufacturing cost of NPs is often higher compared to that of conventional drugs due to the specialized equipment and processes required for production. This makes it more difficult for pharmaceutical companies to justify the cost of development and ensure that the final product is economically feasible. As nanodrugs move from the research phase into clinical trials, the need for cost-effective manufacturing solutions becomes more pressing, especially when considering the financial burden of clinical trials, regulatory approval, and postmarket surveillance.

The regulatory pathway for nanomedicines is still evolving, and regulatory authorities like the FDA and EMA are still establishing guidelines specific to nanodrug delivery systems. NPs often exhibit unique pharmacokinetic profiles, such as prolonged circulation times and targeted accumulation in specific tissues, which require tailored regulatory evaluations. For instance, nanomedicines are subject to more complex toxicological testing than conventional drugs, and the regulatory bodies often require detailed information about the NPs composition, surface properties, and the potential long-term effects of their accumulation in tissues.^161^ In addition, the lack of standardized testing protocols for NPs means that drug developers may face delays in obtaining approval for clinical trials. Nanomedicines are often subjected to additional scrutiny because of potential concerns over their long-term stability and interactions with biological systems, which can also lead to higher development costs. Moreover, designing clinical trials for nanodrugs targeting antiangiogenesis is complicated by the heterogeneity of patients and tumor types. Cancer is not a single disease but rather a collection of many different types of diseases, each with distinct molecular and physiological features. Some tumors may be resistant to antiangiogenesis therapies due to alternative angiogenic pathways, while others may exhibit intratumoral variability in response to therapy. Therefore, clinical trials for antiangiogenic nanodrugs must account for this heterogeneity and often require personalized treatment approaches. Besides, multicenter trials involving diverse patient populations are required to validate the efficacy of nanodrugs. This introduces additional complexities related to patient recruitment, monitoring of adverse effects, and ensuring that treatment responses are accurately measured. Additionally, because antiangiogenesis therapies are often used in combination with other therapies like chemotherapy or immunotherapy, combination therapy design in clinical trials adds further layers of complexity in evaluating the efficacy of these nanodrugs. Overall, the high development and production costs of nanobased drug delivery systems make them economically challenging,^162^ and securing reimbursement for these novel therapies may be difficult, especially when compared to more cost-effective, traditional therapies.

The application of biodegradable and biocompatible materials, like biopolymeric NPs, as well as green chemistry techniques that reduce the formation of toxic components has been driven by safety concerns. The development of NPs that respond to a particular physiological stimulus enhances controlled release mechanisms and enables precise drug administration, which is particularly beneficial in oncology. Additionally, established procedures and cutting-edge techniques like microfluidics can improve the scalability of production processes, guaranteeing constant product quality.^163^ The evolving regulatory landscape requires collaboration to establish clear guidelines for the safety and efficacy evaluation of nanomedicines. To reduce immunogenicity, modifications such as PEGylation and the use of biomimetic materials help NPs evade the immune system. Additionally, optimizing the NP size and shape can enhance tissue penetration, while focused ultrasound techniques can assist in overcoming barriers to drug delivery.^164^ Understanding complex biological interactions through *in vitro* models and advanced imaging techniques aids in refining the NP design. Finally, addressing the high costs of development through streamlined processes and collaborative research can make nanomedicines more accessible.^154^ Overall, these comprehensive strategies highlight the ongoing efforts to overcome the limitations of nanomedicines, paving the way for more effective and safer therapeutic alternatives.^37^

## 8 Conclusions

The development of nanodrugs targeting antiangiogenesis has become a promising approach in the treatment of diseases involving abnormal blood vessel growth, such as cancer.^35^ However, despite the significant potential of these therapies, several challenges and bottlenecks continue to hinder their successful translation into clinical practice. These challenges span across scientific, technical, regulatory, and clinical aspects, each contributing to delays in the adoption of nanodrugs targeting antiangiogenesis. By refining targeted drug delivery, overcoming resistance mechanisms, and harnessing the potential of personalized medicine, the next generation of antiangiogenic therapies holds the promise of more effective and safer cancer treatments, ultimately improving patient survival and quality of life. As research progresses, collaboration between molecular biologists, oncologists, and nanotechnology experts will drive the translation of these innovative approaches from bench to bedside.

## 9 Future Perspectives

The landscape of antiangiogenesis therapy is rapidly evolving, with novel therapeutic targets and innovative delivery mechanisms paving the way for more effective and personalized cancer treatments. Traditional antiangiogenesis therapies have largely focused on targeting VEGF and its receptors; however, resistance mechanisms have highlighted the need for alternative approaches. Emerging targets such as the Notch signaling pathway, metabolic pathways, TME components, and immune system modulation present new opportunities to disrupt tumor angiogenesis.

The Notch signaling pathway regulates EC differentiation and blood vessel maturation. In tumors, dysregulation of Notch signaling often results in disorganized, leaky vessels. Specific inhibitors of Notch signaling, such as γ-secretase inhibitors, have been investigated as strategies to inhibit tumor-induced angiogenesis and improve vascular normalization.^165^ Combining Notch inhibition with traditional anti-VEGF therapy could potentially overcome the resistance to VEGF-targeted approaches and improve therapeutic outcomes.

Another aspect of tumor metabolism that influences angiogenesis is the glycolytic pathway, which is upregulated in many cancer cells to meet the high energy demands. Key enzymes such as hexokinase 2 (HK2) and pyruvate kinase M2 (PKM2) play crucial roles in promoting angiogenesis by generating metabolites that fuel EC proliferation. By targeting these enzymes, it may be possible to reduce the metabolic intermediates that drive angiogenesis and limit tumor vascularization. This approach, known as metabolic reprogramming, has been shown to disrupt EC function and reduce vascular growth in preclinical models.^166^ Furthermore, the EC metabolism can be directly targeted to inhibit angiogenesis. AMP-activated protein kinase (AMPK) plays a critical role in regulating the energy balance of ECs and is involved in processes such as glycolysis and angiogenesis. By inhibition of AMPK, EC proliferation and migration can be suppressed, leading to reduced blood vessel formation. This approach has shown promise in preclinical models as a way to disrupt the metabolic pathways that support angiogenesis.^167^

The TME plays a significant role in supporting tumor growth and angiogenesis, which has led to an increased focus on targeting the TME itself. One promising target is the ECM, which provides structural support to ECs and regulates cell migration. Tumor cells often modify the ECM to promote angiogenesis and invasion. Specific MMPs, which break down ECM proteins, facilitate tumor cell migration and vascularization. Inhibiting MMPs or other ECM remodeling proteins could prevent the pathological remodeling of the TME and reduce blood vessel formation in tumors. Targeting ECM components like fibronectin and collagen may help prevent tumor spread and limit angiogenesis.^168^ Additionally, macrophages in the TME, particularly those polarized into the M2 phenotype, secrete pro-angiogenic factors like VEGF and TGF-β, which promote the formation of new blood vessels. Reprogramming these M2 macrophages into the M1 phenotype, which has antiangiogenic properties, could reduce the pro-angiogenic signals in the TME. Several approaches are under investigation to shift the balance from M2 to M1 macrophages, including the use of small molecules or gene therapies.^169^ Similarly, myeloid-derived suppressor cells (MDSCs) also contribute to tumor-induced angiogenesis by secreting pro-inflammatory cytokines and growth factors. Targeting the interactions between MDSCs and ECs could prevent the development of tumor blood vessels and enhance the effectiveness of other anticancer therapies.^170^

One of the central challenges in cancer therapy is achieving precise targeting of the tumor vasculature to minimize off-target effects and enhance drug accumulation in the tumor. NPs provide an ideal platform for improving targeting specificity due to their ability to be engineered at the nanoscale to interact with specific tumor EC receptors. For example, targeting integrins (e.g., αvβ3, αvβ5) and VEGFRs with NPs has shown promise in directing therapies to ECs involved in angiogenesis.^171^ To improve specificity further, biomarker-driven targeting strategies are emerging. For instance, tumor-associated antigens (TAAs) or EC-specific markers such as CD31 or lectins can be used to guide NPs directly to angiogenic ECs.^172^ Furthermore, targeting via TME-specific features, such as low pH, hypoxia, or overexpressed enzymes (e.g., MMPs), is another avenue that researchers are actively pursuing. Stimuli-responsive NPs can be designed to release their drug payload only in the TME, which minimizes the systemic side effects that are typically associated with cancer therapies.

In the field of gene therapy, CRISPR/Cas9 technology has emerged as a powerful tool for directly modifying genes involved in angiogenesis.^173^ In this sense, CRISPR/Cas9 can be used to knock out genes such as VEGF or HIF-1α. By targeting these genes, CRISPR/Cas9 could effectively halt the angiogenic process at its molecular source, reducing tumor growth and metastasis. Moreover, CRISPR/Cas9 offers a level of specificity that traditional antiangiogenesis drugs may lack. Conventional therapies often work by broadly inhibiting angiogenesis or targeting growth factors but can also affect healthy tissues, leading to side effects. In contrast, CRISPR/Cas9 enables the precise modification of specific genes, minimizing off-target effects and providing a more tailored therapeutic approach. This level of control makes CRISPR a promising option for overcoming the limitations of current antiangiogenesis drugs, potentially leading to more effective and safer therapies for diseases like cancer, where angiogenesis plays a pivotal role in tumor progression. Furthermore, CRISPR/Cas9 can be combined with emerging strategies such as epigenetic editing technologies to provide more precise regulation of angiogenesis.^174^ Epigenetic modifications alter gene expression through mechanisms such as DNA methylation and histone modification. Epigenetic editing tools, such as CRISPR/Cas9-based epigenetic modifiers or other epigenetic enzymes, allow for reversible changes to the epigenome, offering the potential to fine-tune the expression of genes such as VEGF and HIF-1α in a more controlled manner. This approach could provide a more flexible and adaptable method for regulating angiogenesis without permanent genetic alterations, which is particularly advantageous for therapeutic strategies that require the modulation of gene expression rather than gene knockout.

Additionally, noncoding RNAs, including microRNAs and long noncoding RNAs (lncRNAs), have emerged as important regulators of angiogenesis.^175^ MicroRNAs can post-transcriptionally regulate the expression of angiogenic factors like VEGF, HIF-1α, and other signaling molecules, while lncRNAs are involved in modulating various aspects of gene expression, chromatin remodelling, and cellular responses. By targeting these noncoding RNAs with CRISPR/Cas9-based systems, it is possible to regulate the angiogenic process at multiple levels. For example, CRISPR/Cas9 could be used to knock out specific microRNAs that inhibit antiangiogenic pathways or to enhance the expression of lncRNAs that promote the suppression of angiogenesis. Another promising approach is RNA interference (RNAi), which uses siRNAs or antisense oligonucleotides to selectively silence genes involved in angiogenesis. By targeting genes such as VEGF, Notch, or HIF-1α, RNAi therapies can specifically block the signaling pathways that drive angiogenesis, reducing the formation of new blood vessels and potentially enhancing the effectiveness of other cancer treatments.^152^

Gene editing strategies could help fine-tune the angiogenesis process in a way that conventional drugs cannot, providing a new frontier in cancer therapy. However, further research is needed to optimize delivery systems, ensure precision, and fully understand the long-term effects of gene editing in clinical settings. These advancements in gene therapy could open up new avenues for cancer treatment, enabling the development of highly specific therapies that target angiogenesis at its molecular and regulatory roots.^120^

Cancer treatment is often more effective when multiple therapeutic strategies are employed simultaneously. In the case of antiangiogenesis therapy, combining angiogenesis inhibitors with chemotherapy, immunotherapy, or gene therapies can significantly enhance overall therapeutic efficacy. NPs are ideally suited for combination therapy, as they can be engineered to codeliver multiple drugs or genetic materials targeting different pathways involved in tumor progression. For example, NPs can deliver antiangiogenic drugs (e.g., bevacizumab, sunitinib) alongside chemotherapeutic agents (e.g., paclitaxel, doxorubicin) to both inhibit tumor angiogenesis and directly target cancer cells. Nanocarriers can also carry immune-stimulatory agents (e.g., immune checkpoint inhibitors) to enhance antitumor immunity while simultaneously normalizing the tumor vasculature and improving drug delivery.^176^ Immune checkpoint inhibitors such as anti-PD-1 and anti-CTLA-4 can boost the immune response against tumors by blocking immune checkpoint proteins that tumors use to evade immune detection. Combining these inhibitors with antiangiogenic therapies could help normalize the vasculature and enhance immune cell infiltration into the tumor. By improving the delivery of immune cells to the tumor site, this combination approach could result in more effective tumor control.^177^ Additionally, adoptive T cell therapy, where engineered immune cells are transferred into the patient, can benefit from enhanced vascular function when combined with antiangiogenic strategies.^178,179^ Normalizing tumor vasculature can improve immune cell trafficking, making adoptive T cell therapies more effective.

Alternatively, the role of extracellular vesicles in angiogenesis is also gaining attention. Extracellular vesicles are small vesicles secreted by both tumor and ECs that transport pro-angiogenic signals, including growth factors, proteins, and miRNAs. These miRNAs are involved in regulating EC behavior and may enhance tumor vascularization. By targeting the secretion of extracellular vesicles or preventing their uptake by ECs, it may be possible to limit the angiogenic signals that contribute to tumor growth. Additionally, manipulating the cargo of extracellular vesicles to inhibit angiogenesis could present an exciting new therapeutic option for treating cancers with excessive angiogenesis.^180^ In regenerative medicine, stem cells have the potential to restore normal vascular function by generating new ECs and promoting the repair of damaged tissue. Furthermore, stem cells can be engineered to deliver therapeutic agents that inhibit tumor angiogenesis. This dual benefit, repairing damaged vasculature while targeting the tumor’s blood supply, could be particularly useful for cancers with abnormal vascular networks.^181^

The development of hybrid nanocarriers that combine multiple types of nanoparticles—such as polymeric–lipid hybrids or metal–organic frameworks—is also an emerging avenue of interest. These hybrid systems leverage the advantages of different materials to enhance drug loading capacity, stability, and targeting efficiency.^182^ Additionally, nanocarrier systems integrated with artificial intelligence (AI) and machine learning (ML) are being explored to optimize personalized treatment regimens. AI-driven platforms can analyze tumor-specific molecular markers to design tailored NP formulations that deliver precise doses of antiangiogenic agents, increasing the efficiency and success of treatment protocols.^183^

Thus, the future of nanotherapeutic formulations for the delivery of cancer antiangiogenics is greatly promising, with innovative approaches on the horizon to enhance targeting specificity, combination therapies, and personalized treatment strategies. Advances in nanocarrier design, drug release mechanisms, and overcoming resistance will likely lead to more effective anticancer treatments with fewer side effects. By improving the pharmacokinetics, bioavailability, and tumor penetration of antiangiogenic agents, NPs can revolutionize cancer therapy, potentially offering new hope for patients with aggressive or resistant cancers. As nanomedicine formulations continue to evolve, the collaboration among nanotechnology, molecular biology, and clinical oncology will drive the next generation of cancer therapies, ultimately improving patient outcomes and quality of life.

## Data Availability

The authors confirm that the data supporting the findings of this study are available within the article.

## Abbreviations

ES: endostatin
BBB: blood brain barrier
BHE-MDE: N-methyl-2,2′-diaminodiethylamine
CAFs: cancer-associated fibroblasts
CAM: chorioallantoic membrane
CaP: calcium phosphate
CDDP: cisplatin
CPCs: cationic polycarbamates
CpG: unmethylated cytosine-phosphate-guanine
CPP: calcium carbonate-polydopamine-polyethylenimine
cRGD: cyclic arginyl-glycyl-aspartic acid peptide
CS: chitosan
CTLs: cytotoxic T lymphocytes
DC: dendritic cell
DMSC: decidua mesenchymal stromal cells
DOPE: dioleoyl phosphatidylethanolamine
DOTAP: 1,2-dioleoyl-3-trimethylammonium propane
ECM: extracellular matrix
ECs: ECs
EVs: extracellular vesicles
FDA: food and drug administration
FGF: fibroblast growth factor
GCLs: gemini cationic lipids
HAase: hyaluronidase
HA-SH: hyaluronidase-sensitive thiolated hyaluronic acid
HA-Tyr: hyaluronic acid-tyramine
HC: cisplatin-hyaluronic
HCC: hepatocellular carcinoma
HIF: hypoxia-inducible factors
HPSE: heparanase
HREs: hypoxia-responsive elements
HTCS: N-[(2-hydroxy-3-trimethylammonium)propyl] chitosan salt
HUVECs: human umbilical vein ECs
IFN-γ: interferon-γ
IL-12: interleukin-12
IP-10: inducible-protein 10
ISN: immunostimulatory NPs
ITA: itraconazole
IVIS: *In vivo* imaging system
LbL: layer-by-layer
LLC: Lewis lung cancer
MMP: matrix metalloproteinase
MMTV-PyMT: mouse mammary tumor virus-polyoma middle tumor-antigen
MPEG_2000_: (methoxy polyethylene glycol (molecular weight: 2000 g/mol)
mRNA: messenger ribonucleic acid
MSCs: mesenchymal stem cells
NMOFs: nanoscaled metal–organic frameworks
NP: Nanoparticle
NSCLC: nonsmall cell lung cancer
PAMAM: poly(amidoamine)
PAR-1: protease-activated receptor-1
PASp: poly(l-aspartic acid sodium salt)
PBAE: poly(β-amino ester)
PBAE-D: poly(β-amino ester)-dodecyl amine
PCNs: ferritin-based protein cage protein C NPs
PDLLA_4000_: poly(d,l-lactic acid) (molecular weight: 4000 g/mol) PDX: patient-derived xenograft
PECs: pulmonary ECs
PEG: polyethylene glycol
PEG2K-cdm: polyethylene glycol-carboxydimethyl maleate polymer
PEI: polyethyleneimine
pIL-12: plasmid IL-12
PLA: polylactic acid
PLGA: poly(lactic-co-glycolic acid)
PLGF: placental growth factor
Pmet: polymetformin
PS: polyethyleneimine-modified carboxyl-styrene/acrylamide
PTT: photothermal therapy
PTX: paclitaxel
pVAXI-en: secretory endostatin gene
QDs: quantum dots
QDs-M: polymer-quantum dots micelle
R (D)/H(S) NPs: bioresponsive NPs-rotaxane-doxorubicin-heparin-TGF-β receptor inhibitor (SB431542)
RGS16: regulator of G-protein signaling 16
rhES: recombinant human endostatin
SBRT: stereotactic body radiation therapy
SFlt1: soluble fms-like tyrosine kinase-1 (sFlt1)
sFn: small ferritin
shRNA: small hairpin RNA
siRNA: small interfering ribonucleic acid
SNPs: silica biocompatible NPs
SPION: superparamagnetic iron oxide
TAFs: tumor-associated fibroblasts
TAMs: tumor associated macrophages
TAT: transactivating transcriptional activator
TFG: thrombin receptor agonist peptide-small ferritin-PC-Gla
TFMG: matrix metalloproteinase-2 cleavage site is inserted between small ferritin and the PC-Gla domain
tGC: thiolated-glycol chitosan
TGF: transforming growth factor
TGF-β: transforming growth factor beta
Tie2: tyrosine kinase receptor 2
TME: TME
TNF-α: tumor necrosis factor alpha
TPMs: Tie2-positive macrophages
TRAP: thrombin receptor agonist peptide
VEGF: vascular endothelial growth factor
VEGF: vascular endothelial growth factor
α-TOS: tocopheryl succinate
